# Molecular Docking of Detoxification Enzymes from *Oides leucomelaena* with Volatiles of Star Anise

**DOI:** 10.3390/biology14101411

**Published:** 2025-10-14

**Authors:** Yingxue Yang, Zhixiao Zhang, Huifen Ma, Lianrong Hu, Kai Li, Ning Zhao, Ling Liu, Jielong Zhou

**Affiliations:** 1 Forest Resources Exploitation and Utilization Engineering Research Center for Grand Health of Yunnan Provincial Universities, Southwest Forestry University, Kunming 650224, China; yangyingxue1@sina.com (Y.Y.); likaiswfu@163.com (K.L.); lijiangzhn@swfu.edu.cn (N.Z.); 2 College of Biological Science and Food Engineering, Southwest Forestry University, Kunming 650224, China; 3 Yunnan Academy of Forestry and Grassland, Kunming 650224, China; zhangzhixiao@yafg.ac.cn (Z.Z.); mahuifen@yafg.ac.cn (H.M.); hulianrong@yafg.ac.cn (L.H.)

**Keywords:** *Oides leucomelaena* Weise, host adaptability, binding affinity, transcriptome analysis

## Abstract

Star anise, an economically and medicinally important crop, is highly susceptible to infestation by *Oides leucomelaena* Weise, leading to significant yield losses. This study employed transcriptome sequencing and bioinformatic analysis to systematically identify detoxification genes (CYP, GST, and CarE) in *O. leucomelaena* and evaluated their binding potential with star anise volatiles via molecular docking. A total of 64 CYP, 21 GST, and 44 CarE genes were identified, with several genes showing high expression across multiple tissues. Molecular docking revealed strong binding affinity between seven CYP proteins and star anise volatiles, notably the antennae-specific CYP, which exhibited robust binding with multiple volatiles. Detoxification genes in *O. leucomelaena* may mediate host adaptation by metabolizing star anise volatiles, providing potential targets for eco-friendly pest control.

## Simple Summary

Star anise is a valuable crop in China, but it is often severely damaged by a pest (*Oides leucomelaena* Weise). Farmers usually rely on chemical pesticides to control this insect, which can harm the environment. In this study, we aimed to find eco-friendly ways to manage the pest by understanding how it tolerates natural plant defenses. We identified key proteins in the beetle that help it break down chemicals in star anise leaves. Using computer simulations, we found that some of these proteins strongly interact with specific fragrant compounds produced by the plant. This suggests that the beetle uses these proteins to adapt to and survive on star anise. Our findings may help develop new, natural methods to control this pest.

## 1. Introduction

Star anise (*Illicium verum* Hook. F.) is an important traditional spice and medicinal plant in China, with broad application value. In traditional Chinese medicine, it is used to warm yang and dispel cold, regulate qi and relieve pain, and treat digestive system disorders [1,2,3]. Its extracts also possess various pharmacological activities, such as antioxidant, antibacterial, and anti-inflammatory effects [1,4]. Additionally, star anise is a significant economic crop; its essential oil and shikimic acid serve as raw materials for anti-influenza drugs [2,5], leading to high market demand. However, during its growth, the plant is highly susceptible to insect infestations, which can cause plant death and yield reduction, resulting in substantial economic losses [6]. *O. leucomelaena*, one of the major pests of star anise, feeds on the leaves and young shoots as both larvae and adults, causing withering of branches and plant death, which inflicts significant economic damage on regional economies [7]. Current control methods for *O. leucomelaena* primarily rely on chemical pesticides, which are detrimental to environmental protection [8]. At present, research on plant pest control is shifting toward more environmentally friendly approaches.

Insect detoxification genes play a critical role in insect–plant interactions and adaptive evolution [9,10]. These genes serve as key molecular foundations for insects to cope with xenobiotics, such as plant secondary metabolites and insecticides, primarily involving three major enzyme families, namely cytochrome P450 monooxygenases (CYP), glutathione S-transferases (GSTs), and carboxylesterases (CarEs) [11,12,13]. These genes mediate detoxification through metabolic, conjugative, hydrolytic, and efflux mechanisms. Their expression is often regulated by transcriptional factor pathways and can be induced or constitutively upregulated to adapt to varying environmental conditions [14,15]. Generalist insects, such as *Bemisia tabaci* and *Helicoverpa armigera*, can rapidly upregulate specific CYP and GST genes to adapt to different host plants [16,17]. Furthermore, technologies, like RNAi, have demonstrated that targeting detoxification genes can effectively suppress insect resistance, offering novel avenues for pest control [18,19].

Studies have shown that plant secondary metabolites can significantly induce the expression of insect detoxification genes [20]. For instance, multiple CYP6 and GST genes in *H. armigera* are markedly upregulated after feeding on cotton. This induction reflects the chemical interplay between insects and plants shaped by long-term coevolution [16]. Based on these established patterns in other insects, we hypothesize that *O. leucomelaena* will exhibit a similar upregulation of its detoxification gene families (CYP, GST, and CarE) in response to the challenge of star anise volatiles. This is a predictable adaptive response for a specialist pest to overcome host plant chemical defenses. Some plant-derived compounds can inhibit CYP activity, thereby enhancing insecticide efficacy, while the ability of insects to detoxify such compounds may also influence their host-seeking behavior [21]. In summary, the identification and functional analysis of insect detoxification genes provide a theoretical basis for developing behavior-based green pest management strategies. This study aims to investigate the detoxification adaptation mechanisms of *O. leucomelaena* to the volatile compounds of star anise. Through transcriptome sequencing, detoxification gene families (CYP, GST, and CarE) were identified, and molecular docking technology was employed to analyze the binding affinity between key proteins and star anise volatiles, thereby revealing the molecular basis of this pest’s host adaptation at the molecular level. The findings provide a theoretical foundation for developing green control technologies targeting detoxification genes, holding promise for future reductions in chemical pesticide usage and the sustainable management of star anise pests.

## 2. Materials and Methods

### 2.1. Insect Collection and Transcriptome Sequencing

In June 2023, 100 adult specimens of *O. leucomelaena* Weise were collected in Funing County, Wenshan Prefecture, Yunnan Province. Star anise leaves were selected in the laboratory for feeding, and tissue sampling was conducted three days later. In the bioscience laboratory, male and female beetles were dissected and separated, resulting in the successful isolation of tissues including antennae, heads, thoraxes, abdomens, legs, and wings. The tissue was stored at −80 °C. RNA sequencing was performed on these tissues, and annotation of the resulting data has been completed [22]. All experiments involved three biological replicates. Sequencing was performed on the Illumina Novaseq6000 platform (Illumina Inc., San Diego, CA, USA). The raw data, comprising 749 million clean reads, exhibited high quality (Q20: 96.86–98.08%). De novo transcriptome assembly was conducted using Trinity, and the resulting contigs were clustered and refined by removing redundancies with Corset, yielding 171,155 unigenes with an N50 of 1004 bp. All read data are available in the NCBI BioProject database under the project ID PRJNA1123008.

### 2.2. Gene Identification

To identify candidate detoxification genes in *O. leucomelaena*, this study performed a systematic search of the newly obtained independent transcriptome of this beetle, using detoxification gene families from other coleopteran species as queries. The TBLASTN program was employed with an E-value cutoff of 1 × 10^−5^ and an identity of ≥30% to search and preliminarily identify candidate detoxification genes within the *O. leucomelaena* transcriptome. For further validation, the identified genes were verified using TBLASTX against the NCBI non-redundant protein sequence database.

### 2.3. Sequence Analysis and Expression Profiling Construction

Open reading frames (ORFs) were identified using the ORF Finder tool available in NCBI. Multiple sequence alignment was performed with the MUSCLE method in MEGA7.0, and a phylogenetic tree was constructed using the neighbor-joining algorithm. Analysis of the conserved domains in the identified CYPs was performed with ESPript 3.0 (https://espript.ibcp.fr/ESPript/cgi-bin/ESPript.cgi, accessed on 20 September 2025). The resulting tree was subsequently visualized and annotated with iTOL v5. A gene expression heatmap was generated using TBtools v2.362 based on FPKM values (from three biological replicates, log2-transformed and Z-score), defining high expression as a mean FPKM > 10. We selected the MEME Suite (https://web.mit.edu/meme/current/share/doc/overview.html, accessed on 20 September 2025) to perform motif analysis on sequences.

### 2.4. Molecular Docking

To predict the potential function of CYP, we selected highly expressed CYP in the tissue and performed molecular docking simulations between their encoded proteins and volatile compounds from star anise. First, the three-dimensional structures of the target proteins were constructed using the homology modeling server SWISS-MODEL (https://swissmodel.expasy.org/; accessed on 15 March 2025). The modeling process considered sequence similarity to templates, protein homology, and the presence of ligands in the template crystal structures to select the optimal model for subsequent docking analysis. The volatile ligands used for docking were ten compounds previously identified in star anise, as reported in the literature (Supplementary Table S1). These ten compounds were detected in the tender leaves, old leaves, flowers, and fruits of star anise, and are the core common substances in the volatile components of star anise, with representativeness and universality [23]. Molecular docking between proteins and ligands was performed using CB-DOCK [24]. Blind docking is selected for docking, and the docking results are determined through three simulations. If the docking binding energy is less than −7.0 kcal/mol, it can be considered to have strong binding potential.

## 3. Results

### 3.1. Identification and Expression Profile of the oleuCYP Sequence

**Figure 1:**
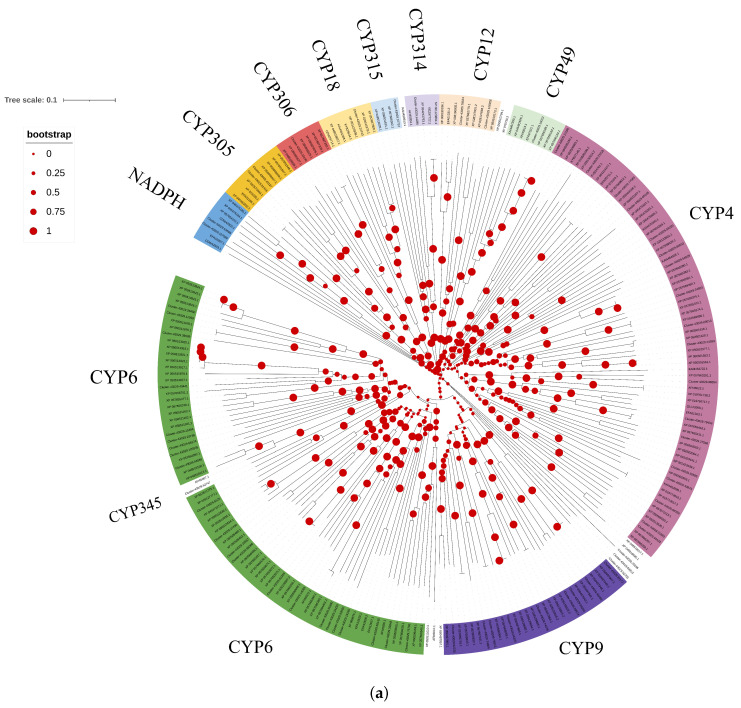

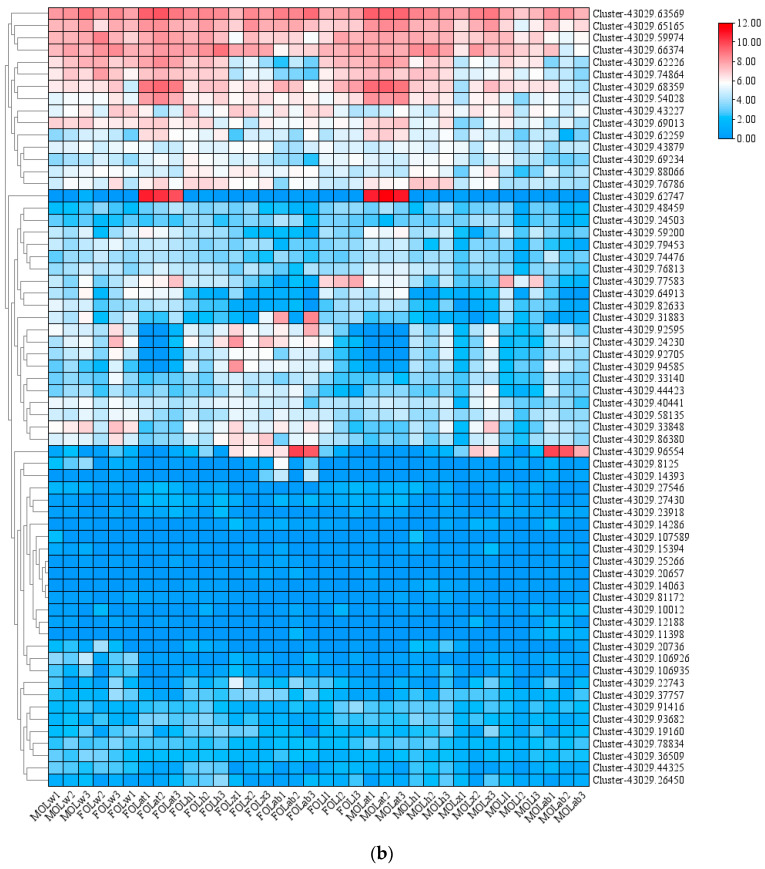
Identification of the OleuCYP family reveals an antenna-specific expressed member. (**a**) Neighbor-joining tree of CYPs. Bootstrap values after 1000 replications; (**b**) expression profiles of CYP genes in *O. leucomelaena*. OL: *O. leucomelaena*. FOLw: female wing; MOLw: male wing; FOLat: female antenna; MOLat: male antenna; FOLh: female head; MOLh: male head; FOLx: female thorax; MOLx: male thorax; FOLl: female leg; MOLl: male leg; FOLab: female abdomen; MOLab: male abdomen.

Through comprehensive analysis, a total of 64 OleuCYP genes were identified. The OleuCYP genes showed high sequence similarity to those from *Diabrotica undecimpunctata* and *Diabrotica virgifera virgifera* (Table 1, Supplementary File S1). Domain analysis confirmed the presence of the heme-binding motif in the CYP proteins (Supplementary Figure S1). According to the CYP nomenclature system, the 64 CYP sequences were classified into distinct families, with the CYP4 family containing 15 proteins and the CYP6 family comprising 22 proteins (Figure 1a). Cluster-43029.62747 belongs to the CYP345 family (Figure 1a). Gene expression heatmap analysis revealed that Cluster-43029.62226, Cluster-43029.59974, Cluster-43029.74864, Cluster-43029.66374, Cluster-43029.63569, and Cluster-43029.65165 were expressed in multiple tissues. Additionally, Cluster-43029.62747 was specifically and highly expressed in antennae (Figure 1b). Screening of high-expression CYPs for subsequent analysis revealed that the motif distribution of CYP proteins is cluster-specific (Supplementary Figure S2).

### 3.2. Identification and Expression Profile of the oleuGST Sequence

**Figure 2:**
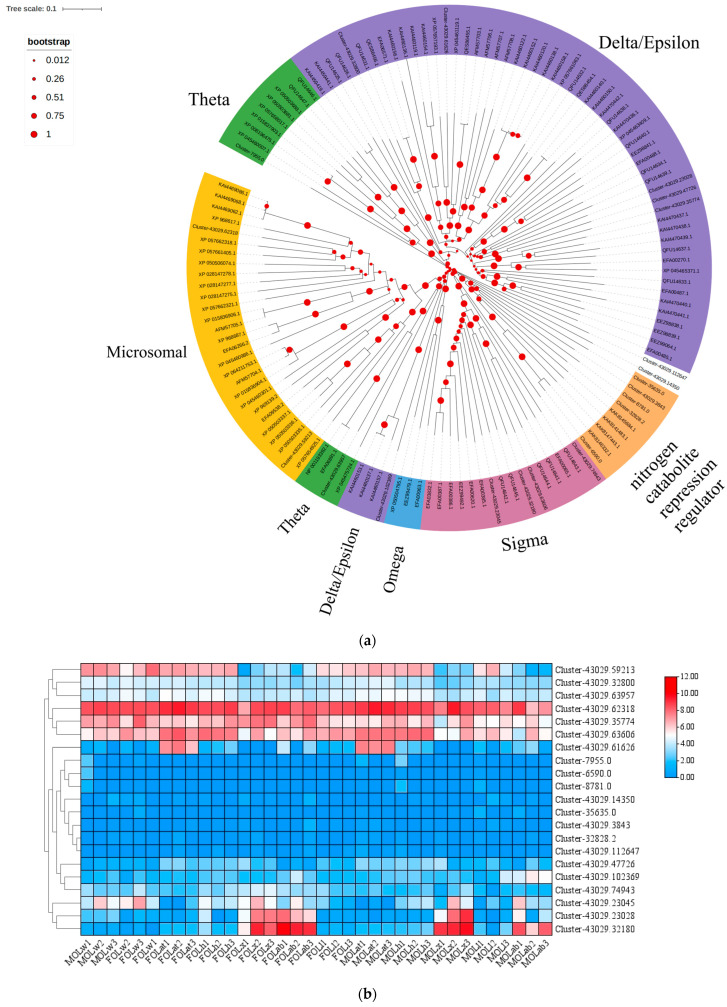
Identification of the OleuGST family. (**a**) Neighbor-joining tree of GSTs. Bootstrap values after 1000 replications; (**b**) expression profiles of GST genes in *O. leucomelaena*. OL: *O. leucomelaena*. FOLw: female wing; MOLw: male wing; FOLat: female antenna; MOLat: male antenna; FOLh: female head; MOLh: male head; FOLx: female thorax; MOLx: male thorax; FOLl: female leg; MOLl: male leg; FOLab: female abdomen; MOLab: male abdomen.

A total of 21 OleuGSTs were identified and analyzed, showing high sequence similarity to those from *Beauveria bassiana* and *Burkholderiales bacterium*, with sequence identity ranging from 54.67% to 100% (Supplementary Table S2, Supplementary File S1). The insect-specific Delta/Epsilon family contains a greater number of GST proteins (Figure 2a). The expression heatmap revealed that Cluster-43029.62318, Cluster-43029.59213, Cluster-43029.35774, and Cluster-43029.63606 were highly expressed in multiple tissues (Figure 2b). Cluster-43029.32180 was highly expressed in the abdomen, while Cluster-43029.23028 was highly expressed in the thorax (Figure 2b).

### 3.3. Identification and Expression Profile of the oleuCarE Sequence

**Figure 3:**
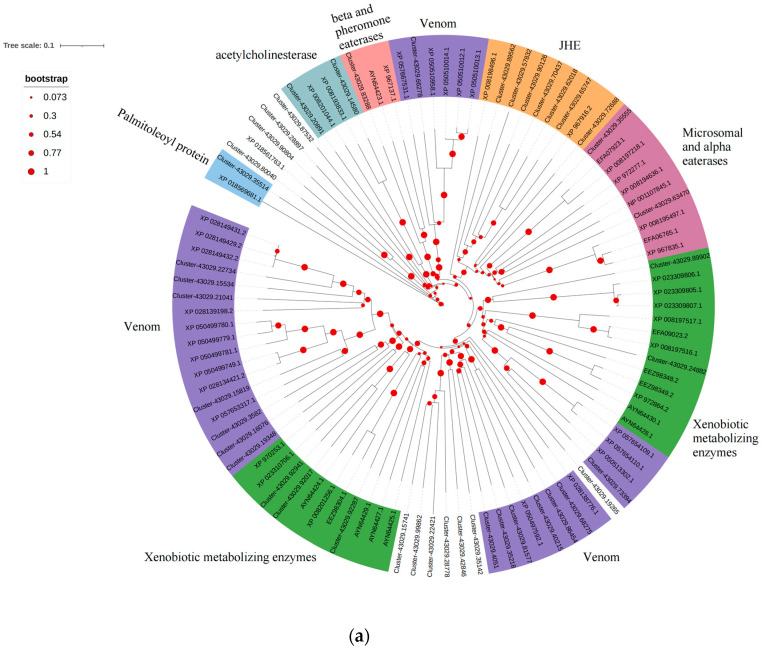

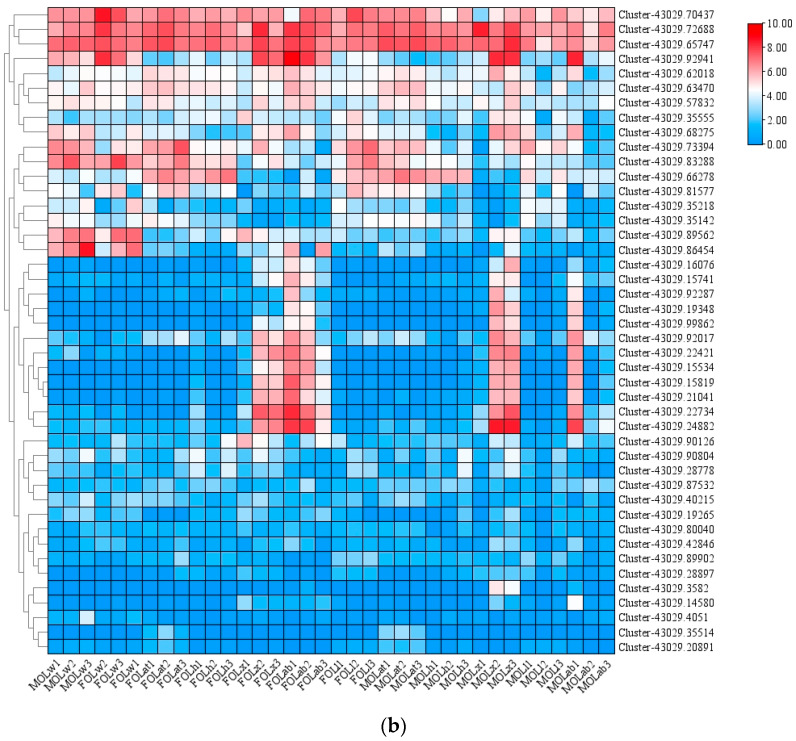
Identification of the OleuCarE gene family. (**a**) Neighbor-joining tree of CarEs. Bootstrap values after 1000 replications; (**b**) expression profiles of CarE genes in *O. leucomelaena*. OL: *O. leucomelaena*. FOLw: female wing; MOLw: male wing; FOLat: female antenna; MOLat: male antenna; FOLh: female head; MOLh: male head; FOLx: female thorax; MOLx: male thorax; FOLl: female leg; MOLl: male leg; FOLab: female abdomen; MOLab: male abdomen.

A total of 44 OleuCarEs were identified, showing high sequence similarity to those from *Leptinotarsa decemlineata* and *Diorhabda sublineata*, with sequence identity ranging from 39.23% to 100% (Supplementary Table S3, Supplementary File S1). Most CarEs belong to the venom family, while CarECluster-43029.89902, Cluster-43029.24882, Cluster-43029.92941, Cluster-43029.92017, and Cluster-43029.92287 belong to the xenobiotic metabolizing enzymes family and are speculated to participate in degrading plant volatile compounds (Figure 3a). The expression heatmap revealed that Cluster-43029.73394, Cluster-43029.65747, and Cluster-43029.83288 were highly expressed in multiple tissues (Figure 3b).

### 3.4. Strong Binding Affinity of Key CYP to Ligands

**Figure 4:**
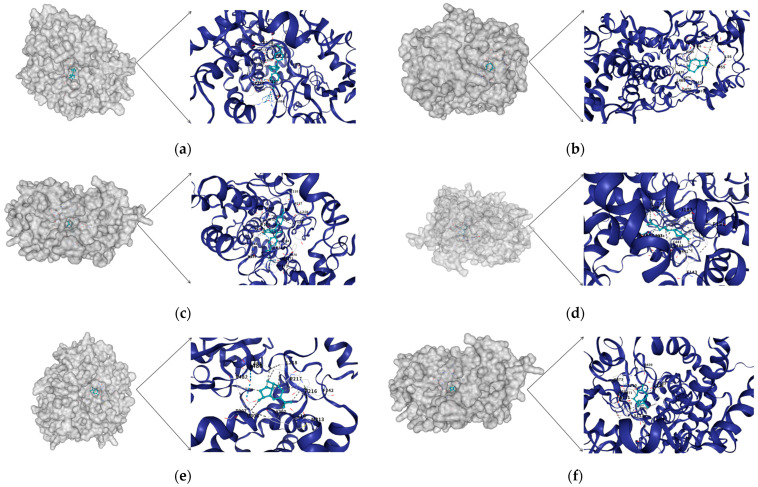
Molecular docking predicts the docking structure between ligands and proteins. (**a**) Cluster-43029.62226 docking results with Anisene; (**b**) Cluster-43029.65165 docking results with β-Caryophyllene; (**c**) Cluster-43029.62747 docking results with Anisene; (**d**) Cluster-43029.62747 docking results with β-Sesquiphellandrene; (**e**) Cluster-43029.62747 docking results with γ-Gurjunene; (**f**) Cluster-43029.62747 docking results with β-Caryophyllene.

Based on gene expression analysis, the proteins encoded by highly expressed CYP genes were selected for molecular docking evaluation. The GMQE range of 7 CYPs is between 0.53–0.87, with a coverage of 0.85–1 and a sequence identity of 30.05–55.51 (Supplementary Table S4). The results indicated that seven proteins exhibited strong binding affinity with all ten ligands (Table 2). Among them, Cluster-43029.62226 showed the highest binding energy with Anisene (Figure 4a), while Cluster-43029.65165 also demonstrated relatively high binding stability with β-Caryophyllene (Figure 4b). Furthermore, Cluster-43029.62747, which is highly expressed in antennae, displayed favorable binding capabilities with multiple ligands, including Anisene, β-Sesquiphellandrene, γ-Gurjunene, and β-Caryophyllene (Figure 4c–f).

## 4. Discussion

This study systematically identified detoxification-related gene families in *O. leucomelaena*, obtaining 64 OleuCYP, 21 OleuGST, and 44 OleuCarE genes. Expression analysis revealed that multiple genes were highly expressed in various tissues, while the CYP gene Cluster-43029.62747 exhibited specific high expression in antennae, suggesting its potential involvement in the perception and initial degradation of volatile compounds. Molecular docking results indicated that multiple CYP displayed high binding affinity with major volatiles of star anise (such as anethole and β-caryophyllene), with Cluster-43029.62226 and Cluster-43029.65165 showing the strongest binding capabilities to anethole and β-caryophyllene, respectively. These results provide clues for a deeper understanding of the detoxification and host adaptation molecular mechanisms of *O. leucomelaena*, and also propose candidate targets for developing green prevention and control strategies based on behavioral interference.

CYPs are a class of enzymes widely found in insects, primarily responsible for the metabolism of xenobiotics, including the detoxification of insecticides [25]. In coleopteran pests, the CYP gene family is typically large, which is associated with their broad metabolic functions [26]. The expression levels of these genes may vary across different tissues and are significantly regulated by environmental factors [27,28,29]. Among coleopteran insects, the number of identified CYP genes shows remarkable interspecies variation, and their functions are primarily focused on detoxification metabolism and hormone regulation. Specifically, *Tribolium castaneum* possesses 143 CYP genes, with representative genes CYP6BQ7 and CYP4G7 confirmed to be involved in insecticide metabolism [30,31]. In contrast, 4 species of Tenebrionidae exhibit varying numbers, with 103 in *Tenebrio molitor*, 157 in *Asbolus verrucosus*, 122 in *Hycleus cichorii*, and 101 in *Hycleus phaleratus* [32]. The expansion of these genes is believed to be closely related to species adaptation to chemical environments. The *L. decemlineata* has been found to have 74 CYP genes, among which genes, like CYP12H2, are associated with insecticide detoxification [33]. Among the 64 CYP genes identified in the *Dendroctonus armandi*, members of the CYP4 family were revealed to oxidize monoterpenoid defensive compounds from host trees, thereby enhancing ecological fitness [34,35]. Research on the *Aethina tumida* has confirmed that its CYP genes are involved in ecdysteroid metabolism [36]. The CYP4 family genes in *D. virgifera* exhibit insecticide susceptibility [37]. This study identified 64 CYP genes in *O. leucomelaena*, with significant interspecies differences in their numbers, which may reflect different adaptation strategies of species to ecological and chemical environments. Overall, these functionally diverse CYP genes, classified within an evolutionary clade framework, constitute the core molecular foundation for coleopteran insects to respond to environmental toxins and maintain physiological homeostasis through the expansion and differentiation of gene families [38,39].

GSTs are another important class of detoxification enzymes, primarily responsible for conjugating glutathione with toxic compounds to facilitate their excretion [40,41]. In coleopteran pests, the number of GST gene family members is relatively small, but they exhibit high functional diversity. GST gene families in coleopteran insects show significant species-specificity and diversity in both number and function. Gene counts vary markedly among species: *T. castaneum* has 36 cytosolic and 5 microsomal GSTs [42], *Sitophilus oryzae* possesses 26 [43], while *D. armandi* was found to have 9 new genes [44]. The core function of GST genes lies in detoxification metabolism, where they have been directly proven to metabolize various insecticides. Their knockout or inhibition significantly increases insect susceptibility to insecticides [45,46]. Additionally, they play key roles in developmental regulation and stress response.

CarEs in coleopteran insects exhibit striking species-specific diversity in both gene number and function. Gene counts vary substantially among species: *T. castaneum* and *T. molitor* possess over 60 and 53 CarE genes, respectively, while *A. verrucosus* contains up to 105 genes, primarily due to the expansion of α-esterases [47]. In contrast, only 8 CarE genes were identified in *D. armandi* [48]. CarEs play crucial roles in detoxification metabolism, with CarEs of *D. armandi* degrading plant defensive terpenoids [49] and CarEs of *T. castaneum* hydrolyzing various insecticides [50]. They also participate in physiological regulation through juvenile hormone esterase-mediated degradation for developmental timing, acetylcholinesterase-mediated neurotransmitter hydrolysis for neural signaling, and potential pheromone degradation [51,52,53]. This functional diversity is reflected in tissue- and developmental stage-specific expression patterns, demonstrating the gene family’s core evolutionary role in ecological adaptation and chemical defense regulation.

This study systematically identified detoxification-related genes in *O. leucomelaena*. Expression profiling revealed tissue-specific expression patterns, with CYP gene Cluster-43029.62747 specifically highly expressed in antennae, while molecular docking simulations predicted high binding affinity between multiple CYP proteins and major volatiles of star anise. These findings collectively form a scientific hypothesis that these CYP genes may be involved in the perception and metabolic detoxification of host plant volatile compounds. However, it must be clearly stated that a key limitation of this study is that its conclusions are entirely based on bioinformatic predictions and lack functional experimental validation; therefore, the actual metabolic functions of these CYP genes remain unconfirmed. Based on this, we propose a phased future validation plan: first, selected CYP genes will be co-expressed with insect CYP reductase and the protein complexes purified, followed by in vitro enzymatic assays combined with LC-MS technology to directly detect metabolites of star anise volatiles; after obtaining in vitro confirmation, in vivo RNAi or gene editing experiments will be conducted to ultimately verify their physiological functions at the organismal level. Subsequent research aims to transform the current predictive evidence into conclusive functional insights.

## 5. Conclusions

This study systematically identified and characterized three major detoxification gene families (CYP, GST, and CarE) in *O. leucomelaena* through transcriptomic analysis. The discovery of 64 CYP, 21 GST, and 44 CarE genes, along with their tissue-specific expression patterns, provides a molecular basis for the beetle’s adaptation to star anise. Notably, molecular docking demonstrated strong binding potential between key CYP enzymes and volatile compounds from star anise, suggesting that these genes may be involved in metabolizing plant defensive chemicals—though this remains preliminary and requires experimental validation. These findings offer valuable clues for developing targeted and eco-friendly pest management strategies.

## Figures and Tables

**Table 1:** OleuCYP gene identification information.

ID	Name	ORF (aa)	Scientific	E Value	Per Ident	Accession
Cluster-43029.107589	OleuCYP-NADPH	595	*Beauveria bassiana*	0	99.66%	PMB72824.1
Cluster-43029.63569	OleuCYP-NADPH	272	*Diabrotica undecimpunctata*	0	90.44%	XP_072381184.1
Cluster-43029.22743	OleuCYP18a1	528	*Diabrotica virgifera virgifera*	0	88.45%	XP_028141979.1
Cluster-43029.10012	OleuCYP49a1	544	*Diabrotica undecimpunctata*	0	84.71%	XP_072391690.1
Cluster-43029.8125	OleuCYP6k1	499	*Diorhabda carinulata*	0	81.26%	XP_057671775.1
Cluster-43029.14063	OleuCYP6a14	515	*Diabrotica undecimpunctata*	0	78.29%	XP_072379934.1
Cluster-43029.25266	OleuCYP302a1	291	*Diabrotica virgifera virgifera*	9.00 × 10^−140^	71.13%	XP_028142564.1
Cluster-43029.79453	OleuCYP4d2	505	*Diorhabda carinulata*	0	75.30%	XP_057655410.1
Cluster-43029.43227	OleuCYP305a1	448	*Diabrotica virgifera virgifera*	0	75.58%	XP_050508447.1
Cluster-43029.96554	OleuCYP4g15	557	*Diabrotica undecimpunctata*	0	75.85%	XP_072385487.1
Cluster-43029.33848	OleuCYP6a8	518	*Diabrotica virgifera virgifera*	0	75.05%	XP_050508500.1
Cluster-43029.78834	OleuCYP12a5	354	*Diabrotica virgifera virgifera*	2.00 × 10^−171^	65.92%	XP_028147894.2
Cluster-43029.88066	OleuCYP6a13	517	*Diabrotica virgifera virgifera*	0	71.48%	XP_050508496.1
Cluster-43029.43879	OleuCYP4c1	494	*Diabrotica undecimpunctata*	0	71.25%	XP_072382925.1
Cluster-43029.62226	OleuCYP9e2	525	*Diabrotica virgifera virgifera*	0	68.38%	XP_050497957.1
Cluster-43029.33140	OleuCYP306a1	510	*Diabrotica undecimpunctata*	0	69.74%	XP_072388304.1
Cluster-43029.59200	OleuCYP4aa1	471	*Diabrotica undecimpunctata*	0	70.70%	XP_072396269.1
Cluster-43029.76786	OleuCYP6a2	396	*Diabrotica virgifera virgifera*	0	68.10%	XP_050509163.1
Cluster-43029.62259	OleuCYP6bj70	421	*Monolepta hieroglyphica*	0	65.95%	XHH54104.1
Cluster-43029.74476	OleuCYP9e2	528	*Diabrotica undecimpunctata*	0	65.57%	XP_072396671.1
Cluster-43029.68359	OleuCYP4c1	458	*Diabrotica undecimpunctata*	0	66.22%	XP_072390792.1
Cluster-43029.48459	OleuCYP9e2	532	*Diabrotica virgifera virgifera*	0	65.69%	XP_050497942.1
Cluster-43029.81172	OleuCYP9e2	513	*Diabrotica virgifera virgifera*	0	64.58%	XP_050497945.1
Cluster-43029.59974	OleuCYP9e2	523	*Diabrotica undecimpunctata*	0	64.05%	XP_072397933.1
Cluster-43029.91416	OleuCYP305a1	493	*Diorhabda carinulata*	0	63.69%	XP_057671993.1
Cluster-43029.69013	OleuCYP4d14	499	*Diabrotica virgifera virgifera*	0	61.69%	XP_050501618.1
Cluster-43029.37757	OleuCYP315a1	462	*Diabrotica undecimpunctata*	0	66.31%	XP_072382045.1
Cluster-43029.14393	OleuCYP4c1	496	*Diabrotica undecimpunctata*	0	63.77%	XP_072402566.1
Cluster-43029.40441	OleuCYP6k1	503	*Diorhabda sublineata*	0	60.04%	XP_056644762.1
Cluster-43029.92595	OleuCYP4c1	488	*Diabrotica undecimpunctata*	0	66.60%	XP_072382925.1
Cluster-43029.69234	OleuCYP9e2	524	*Diabrotica virgifera virgifera*	0	61.83%	XP_028133915.2
Cluster-43029.12188	OleuCYP4c3	481	*Diabrotica undecimpunctata*	0	61.20%	XP_072379783.1
Cluster-43029.74864	OleuCYP9e2	502	*Diabrotica virgifera virgifera*	0	61.43%	XP_050497948.1
Cluster-43029.54028	OleuCYP4bn80	522	*Monolepta hieroglyphica*	0	64.44%	WKR34928.1
Cluster-43029.76813	OleuCYP9e2	502	*Diabrotica virgifera virgifera*	0	60.64%	XP_050497948.1
Cluster-43029.24503	OleuCYP4d	498	*Diabrotica undecimpunctata*	0	53.04%	XP_072402574.1
Cluster-43029.14286	OleuCYP314a1	490	*Colaphellus bowringi*	0	72.39%	UYL69089.1
Cluster-43029.20657	OleuCYP6a23	455	*Diabrotica undecimpunctata*	0	62.72%	XP_072392648.1
Cluster-43029.44423	OleuCYP4c1	507	*Diorhabda carinulata*	0	59.21%	XP_057666257.1
Cluster-43029.64913	OleuCYP	531	*Leptinotarsa decemlineata*	0	48.77%	AAZ94269.1
Cluster-43029.106926	OleuCYP12a2	337	*Diabrotica virgifera virgifera*	2.00 × 10^−138^	57.96%	XP_050510784.1
Cluster-43029.27430	OleuCYP6a2	520	*Diabrotica virgifera virgifera*	0	58.11%	XP_050512634.1
Cluster-43029.86380	OleuCYP6a20	497	*Diorhabda sublineata*	0	57.75%	XP_056634040.1
Cluster-43029.44325	OleuCYP9e2	527	*Diabrotica undecimpunctata*	0	57.58%	XP_072390190.1
Cluster-43029.31883	OleuCYP	506	*Agasicles hygrophila*	0	62.08%	AZR39463.1
Cluster-43029.15394	OleuCYP6k	494	*Diabrotica virgifera virgifera*	0	57.40%	XP_050511921.1
Cluster-43029.77583	OleuCYP	571	*Agasicles hygrophila*	0	59.27%	AZR39479.1
Cluster-43029.58135	OleuCYP6a	501	*Anoplophora glabripennis*	4.00 × 10^−178^	49.60%	XP_023310525.1
Cluster-43029.26450	OleuCYP6k	507	*Diabrotica virgifera virgifera*	0	56.02%	XP_050513923.1
Cluster-43029.82633	OleuCYP4v	482	*Diorhabda sublineata*	5.00 × 10^−160^	46.06%	XP_056640038.1
Cluster-43029.62747	OleuCYP345h	499	*Monolepta hieroglyphica*	0	63.47%	XHM34208.1
Cluster-43029.65165	OleuCYP	489	*Agasicles hygrophila*	2.00 × 10^−154^	46.75%	AZR39465.1
Cluster-43029.92705	OleuCYP	490	*Pharsalia antennata*	0	50.96%	WCC58103.1
Cluster-43029.11398	OleuCYP6k	420	*Diabrotica virgifera virgifera*	2.00 × 10^−164^	53.83%	XP_050513923.1
Cluster-43029.23918	OleuCYP4c	506	*Diabrotica virgifera virgifera*	2.00 × 10^−127^	39.60%	XP_028132814.2
Cluster-43029.24230	OleuCYP6a	499	*Diorhabda sublineata*	0	52.51%	XP_056634040.1
Cluster-43029.66374	OleuCYP6k	347	*Diorhabda sublineata*	4.00 × 10^−128^	53.62%	XP_056633285.1
Cluster-43029.27546	OleuCYP4c	489	*Diabrotica virgifera virgifera*	4.00 × 10^−176^	50.72%	XP_050502063.1
Cluster-43029.36509	OleuCYP6	496	*Diabrotica undecimpunctata*	2.00 × 10^−135^	39.31%	XP_072393601.1
Cluster-43029.94585	OleuCYP6k	500	*Diorhabda sublineata*	0	50.72%	XP_056633285.1
Cluster-43029.93682	OleuCYP6a	496	*Diorhabda sublineata*	3.00 × 10^−180^	48.90%	XP_056634040.1
Cluster-43029.106935	OleuCYP6k	496	*Diorhabda sublineata*	2.00 × 10^−175^	48.58%	XP_056633285.1
Cluster-43029.20736	OleuCYP6k	495	*Diabrotica virgifera virgifera*	1.00 × 10^−167^	47.98%	XP_050511921.1
Cluster-43029.19160	OleuCYP6a	492	*Diabrotica virgifera virgifera*	2.00 × 10^−175^	47.76%	XP_028130066.2

**Table 2:** Key CYP and compound molecular docking results display. Data presented as binding energy (Kcal/mol).

	Cluster-43029.59974	Cluster-43029.62226	Cluster-43029.62747	Cluster-43029.63569	Cluster-43029.65165	Cluster-43029.66374	Cluster-43029.74864
γ-Gurjunene	−6.6	−6.4	−7.2	−7.1	−7.7	−7.2	−7.5
β-Caryophyllene	−6.7	−6.9	−7.2	−7.1	−8.0	−6.7	−7.5
β-Elemene	−6.3	−5.9	−6.4	−6.8	−7.1	−7.9	−6.5
γ-Elemene	−6.6	−6	−6.5	−6.9	−7.5	−7.5	−6.6
Anisene	−7.3	−8.7	−7.7	−7.5	−7.1	−7	−7.4
Anethole	−6.2	−6	−6.2	−6	−6	−5.7	−6.0
Foeniculin	−7.1	−7.5	−7.2	−6.9	−7.1	−6.9	−7.2
β-Sesquiphellandrene	−6.7	−6.3	−6.4	−7.4	−7.2	−7	−7.6
a-Farnesene	−6.4	−5.9	−6.7	−6.6	−7	−6.9	−7.1
Estragole	−5.8	−5.6	−6.3	−5.9	−5.7	−5.6	−5.9
γ-Gurjunene	−6.6	−6.4	−7.2	−7.1	−7.7	−7.2	−7.5

## Data Availability

All read data are available in the NCBI BioProject database under the project ID PRJNA1123008.

## Supplementary Materials

The following supporting information can be downloaded at: https://www.mdpi.com/article/10.3390/biology14101411/s1, File S1: Detoxification protein sequence of *O. leucomelaena*; Figure S1: OleuCYP sequence alignment; Figure S2: Motif analysis of key CYPs; Table S1: Compound information; Table S2: OleuGST identification information; Table S3: OleuCarE identification information; Table S4: OleuCYP modeling information.
