# Supplementary material for: Molecular Docking of Detoxification Enzymes from Oides leucomelaena with Volatiles of Star Anise
Source: Biology (Basel). 2025 Oct 14;14(10):1411. doi: 10.3390/biology14101411 (PMC12562175; doi:10.3390/biology14101411)
Supplement: Supplementary file 1 [file biology-14-01411-s001.zip › Supplementary Figure S1.pdf]

```
                                                    1        10        20        30
Cluster-43029.107589   ..............................MVADLEDYDYENLDEFPSDKIIMFVLATYG
Cluster-43029.63569    ............................................................
Cluster-43029.22743    .............................MFMYTASVLWDYTKQEFTTNILFVFFTVLLV
Cluster-43029.10012    ......................MSRQVSRKLTITTIQNCRQYSTDRPYSTALNVVSEENQ
Cluster-43029.8125     .................................MSTMQVSTNWSSLALLVVALYLFYKYV
Cluster-43029.14063    ...............................MELLTR.....NVAAAILFALFLLLYNYY
Cluster-43029.25266    ................................MNFCR.....KNNCVTNCILKERKKYFI
Cluster-43029.79453    .........................................MWMVSVIFGAVVIFITTLL
Cluster-43029.43227    ..........................................MIMFTVLVGLVILIIFFV
Cluster-43029.96554    ........................MSQPVLIPAAVPSVIGTPLQLALVGIATILALYQYW
Cluster-43029.33848    ...............................MISYTT.....TCVLSIAVVFITYIIRLF
Cluster-43029.78834    ............................................................
Cluster-43029.88066    ..................................MAA.....VTVFYVTIALVILVYIYL
Cluster-43029.43879    .................................MIWL.....ILLFVVLVLVALFIKRRK
Cluster-43029.62226    ........................................MLVLILIIV.GVAVVFYNFA
Cluster-43029.33140    .............................MVSWCFDLNDFKMVYIYLIAGALIWVFYTI.
Cluster-43029.59200    ................................MDYYL.....GYFISIILAIFSIIKNYI
Cluster-43029.76786    ............................................................
Cluster-43029.62259    ............................................................
Cluster-43029.74476    ........................................MVEIIFLIVGGVGAYIYFCI
Cluster-43029.68359    ..............................MEHLVEP.....IFFAIATVIVVYCVYKYF
Cluster-43029.48459    .................................MFTM.....LVFIFLAILSLILSYYFI
Cluster-43029.81172    ........................................MSSLLILIVICCCCLLYWYV
Cluster-43029.59974    .................................MF.........WILGAILLITLFYYFG
Cluster-43029.91416    ...........................................MLVLIVEAIFALLFIYL
Cluster-43029.69013    ...................................MISLIC.AVTVLGFLLLIILHKRYV
Cluster-43029.37757    ..............................................MFAKKLVKISRHFR
Cluster-43029.14393    ..........................................MLTLIFIAALLVLWYVYV
Cluster-43029.40441    .............................MILTSSWI.....VDFLLFSSVGCFLIYKYV
Cluster-43029.92595    ...........................................MFLLLAIIIPLLVYYII
Cluster-43029.69234    .................................MF.........WILVGILLVTIFYYFG
Cluster-43029.12188    ...........................................MILFITFVLILLIHYYV
Cluster-43029.74864    ............................................................
Cluster-43029.54028    ................MTLVGANKLKEMMSLSTNSHKLVFSLEVFIVTTLVIVVTYILYQ
Cluster-43029.76813    ............................................................
Cluster-43029.24503    .........................................MGLLLTIAFIVFTISYVLY
Cluster-43029.14286    ...............................MINELF.....YTFSTFNCIIVIVVFLIV
Cluster-43029.20657    ............................................................
Cluster-43029.44423    ................................MILVA.....CVVFLIVIITCLYATRSI
Cluster-43029.64913    .................................MVFN.....ALLLTCIVSAILLISYYC
Cluster-43029.106926   ............................................................
Cluster-43029.27430    ...................................ML.....FVFVWMIAALPVLLYIFV
Cluster-43029.86380    .............................MFVTSHGF.....YDISVVIVTFIVISVFYV
Cluster-43029.44325    ..........................................MIPLVLIGIVIALFYYVA
Cluster-43029.31883    ..................................MSIWYFCTYFLLILFLTSILVLKKLK
Cluster-43029.15394    .............................MLLSSSWT.....VDFGILLLSGIVLLYSYI
Cluster-43029.77583    MSSICTVTQSFIKVLNLTGTVGNPKRLAGGPNVYTSQKISKSLLLTIGLILLIWYVQYLW
Cluster-43029.58135    ............................MGYFLDSTV.....VDLLIVLTTVLVSVFMYF
Cluster-43029.26450    .............................MLITPSWT.....LDLLLVVSVVIYLLYKYF
Cluster-43029.82633    ..........................................MYCLIYFAITSFLILIYL
Cluster-43029.62747    .............................MLLTSLWI.....VDIGLFVMIIVFSLYKYS
Cluster-43029.65165    ..........................................MSVLIFLLTVIANFFLYW
Cluster-43029.92705    ....................................................MMIRCRLV
Cluster-43029.11398    ............................................................
Cluster-43029.23918    ............................MDNNKSSEYTSFGILFFPLAITIFIYLIQLLW
Cluster-43029.24230    .............................MLFSNTIL.....CNISIFCTTVCIITYTYI
Cluster-43029.66374    ............................................................
Cluster-43029.27546    .........................................MFLVIILLLLLLAYYFNRR
Cluster-43029.36509    ..............................MILTYVG.....ISTMLLLPTVAVLLYFYM
Cluster-43029.94585    ..............................MLETSTI.....AYLTIVIGLLLLLFYRYM
Cluster-43029.93682    .............................MLVSSNFS.....TDVCILLATLSIIGYFYI
Cluster-43029.106935   .................................MMLLTTSLPTDVTILLGLLLCLLYIYI
Cluster-43029.20736    .............................MIFTSSWI.....VDIVFFGILGMYLSKKYA
Cluster-43029.19160    .........................................MTNILLFLIFIVTFIFFYA
```

```
                                                   40             50                 60
Cluster-43029.107589  EGEPTD....................NAVEFYEHIRGDDVSFT....NGSSLENLKYVA
Cluster-43029.63569   ...........................................................M
Cluster-43029.22743   VRALQM...LKET.........ISLPPGP.WGLP.IMGSLPFVK.........GDLHLHY
Cluster-43029.10012   EKDIFE...YKVENVDRIPKEYKDIP.GP.KELP.FIGNSWRFA.PIIGHYKIHELDKVM
Cluster-43029.8125    TRNHNY...WKRR.........GVP.YE.EPFF.LAGNFWEVF...SGKMQIGKHLGHL
Cluster-43029.14063   RNAFKY...WRNI.........KVP.QLDPRFP..FGDMYETI...LRKQNMGDKIKEI
Cluster-43029.25266   KKYLHS...FE...........NIP.GP.KSLP.AIGTLYQYL.PFIGKYRFDELHYNG
Cluster-43029.79453   YWYFSL...WKYS.......SLENVA.GP.KPLP.IIGNAN......LVGKTTVSLLESF
Cluster-43029.43227   ....SF...KKPK.........NYPPGP.IWYP.IVGNFYMLKRLSKLSGSQHKAFEKL
Cluster-43029.96554   FKTQRY...VKMG........NKIP.GL.PTIP.FFGNAHLAI..GKSASDIFDFAMDM
Cluster-43029.33848   HNSFQY...WKNK.........NVP.YLKPTII.PLGNTLDLY..LGKVTFGELFSNA
Cluster-43029.78834   .MKNAV...KSFM.........DIP.TL.RSLP.LVQHTYLFF..PGGKYKSERLTEAV
Cluster-43029.88066   KWNFTY...WKRK.........GLE.QI.EPIHIIFGNTKGFV...NKKLSFGDQLTNL
Cluster-43029.43879   KTYNEI...WKYL.........DAIPEP.KSYP.ILGTNHVLH.......TPESMFIRD
Cluster-43029.62226   IKPMSY...WKER.........GVK.QG.KAYW.LVGNNLSTI...IGTKSFLEATLDI
Cluster-43029.33140   .........WLNS.........RLPPGP.WGLP.IFGYLLWLD........PKNPYVTL
Cluster-43029.59200   RSVILV...WSMK.............GP.PALP.ILGNALLLK.DNDALLQYGDHAHTL
Cluster-43029.76786   ...........................................................
Cluster-43029.62259   ..........................MP...............................
Cluster-43029.74476   IKPWQY...WKDR.........NVD.QA.TISY.ILKNSYNFF...AKKISMAEANVEG
Cluster-43029.68359   KLRNVL...TKID.........KIP.GP.DKVP.ILGNLLEIL.....KCDTVQLFEKF
Cluster-43029.48459   FVPHNF...WKTR.........GVK.QK.GILP.VFIQSWFSL...FKRHSVGDMVKGL
Cluster-43029.81172   VWLQRY...WKRK.........GIQ.QK.NTVW.LFGDLFWYM...LRRESTADLVKRV
Cluster-43029.59974   IKPLFY...WKER.........GVK.QD.DLWT.LLFSNWFSL...FRRLTFYEFANEL
Cluster-43029.91416   LLEIKK...PINY.............PPGPIWYPMVGAVPTLK...NLSKSRGLYLAIL
Cluster-43029.69013   NNIQKY...LK...........DVA.GP.TPNP.IFGNMLKFA......VPTHKFLDVI
Cluster-43029.37757   SFSIGK...FE...........DIP.SP.IGLP.LIGTTFSLL....ASGGPKKLHKYM
Cluster-43029.14393   SSRRNT...KYLK.........DIP.GP.KPNV.FIGNMDDFM.....FIPNHKYFDVL
Cluster-43029.40441   TRHFSY...WKKR.........GVY.YP.QPIP.FFGNFYDVL...TLKTTIQEHMKKY
Cluster-43029.92595   KRKRQYDHIWKHL.........ELIPSP.YSIP.ILGNIYKLH.......TQESLFIRG
Cluster-43029.69234   LKPLTY...WKDK.........GVK.QP.EILT.AFLENWLTI...FKAISIVEFIKQR
Cluster-43029.12188   NRKLFW.LSWKLS.............GP.TALP.IIGNALTFL......CNNDDLLERI
Cluster-43029.74864   ...MRY...WKER.........GIK.QT.KPVW.FLGDNWGTL...IRQHSLVDFIQHV
Cluster-43029.54028   KIRNRYYLNWIPA.............APAPSI..ILGHAPELL.......DPTETLKSL
Cluster-43029.76813   ...MRY...WKER.........GIK.QT.KPVW.FLGDKWGTI...IRQHNMVDFIQHV
Cluster-43029.24503   KKYINS...KYMK.........NVP.GP.PSNF.FFGNLFYVL.....LRPKHEHLNIL
Cluster-43029.14286   GYRPPW...WNKKAIGCARKEIQDIP.GP.LSLP.LLGTRWVFT...FGGYKFNKIHEFY
Cluster-43029.20657   ......................................................MLNEL
Cluster-43029.44423   KKRVRF...IKLL.........STLPGP.EQKH.LLGNLIDLR...AKPTTFFKHIRAW
Cluster-43029.64913   IRSHNY...WKNK.........RIL.QE.KSTL.LFGDAFLYI...FGIESFAELLLRL
Cluster-43029.106926  ...........................................................
Cluster-43029.27430   KWRHSF...WLRK.........GVV.QIEPEFI...KGNTGNV...RTKTHLNIVIQKL
Cluster-43029.86380   NYKYTY...WKRK.........GII.QLKPTFP..TGNYARNF...PRGISIGGITKKY
Cluster-43029.44325   IKPLSY...WKDR.........GVI.QG.DPVW.LLGDTWRCM...LGLESFADMIQRG
Cluster-43029.31883   NRIVST...ILIN.........KLP.SP.KGLP.ILGHFFVIL......LDDDMFFKNC
Cluster-43029.15394   SSKQKY...WKKR.........GVL.TP.KIQP.IFGHFIDIV...LGRTTIAELLKRY
Cluster-43029.77583   KRRRLYILSWNIP.............GP.FAFP.FIGAAYCFL.....GSETSDIMEAI
Cluster-43029.58135   QYSVKY...WERR.........NIP.SLNQGFPKFVG.........GRSLHFSLVHKVY
Cluster-43029.26450   TRHYDH...WKQL.........GIY.SP.KPIP.FFGNVYDIF...TFKTTIAKGLKDL
Cluster-43029.82633   IRRNLK...HLDA.............IPMPPCNVISGHIFTLF.......KPTEILDKI
Cluster-43029.62747   TRKFDY...WKKR.........DVP.YD.KPVP.FFGNFKNVL...LLKTTVGEWLQKS
Cluster-43029.65165   CYDIYR...KNKL.......LEKIP.GP.PPIP.FFGM........KLPSDIYGFHKML
Cluster-43029.92705   KRHVDY...FVKC.............TY......IVNIFYSIR................
Cluster-43029.11398   ...........................................................
Cluster-43029.23918   SRRKLYLCSWKLP.............GN.VAFP.IIGHAHKFM.....FCTRVGILKFI
Cluster-43029.24230   KWKYGY...WKRK.........GVV.QLQPVFP..FGNYFKAL...PRGVGLGYVTKTF
Cluster-43029.66374   ...........................................................
Cluster-43029.27546   RKNDEF...WKRF.........MAIPGP.KGMP.IIGTLPISTDEEERWLEFGSFSKQF
Cluster-43029.36509   IRNFQY...WKKK.........GVP.YI.PPVP.FFGNYYKMI...TYRTSMAVGFKQL
Cluster-43029.94585   SIKQQY...WQKK.........GVY.TP.KTVP.IIGHFLDVA...LFRCSMGEWLRKY
Cluster-43029.93682   KWKYGY...WKRR.........GIA.QLNPVFP..LGDLHLAL...PRGPIIGYLSKKF
Cluster-43029.106935  ARSHQY...WQKK.........RVH.TP.KIVP.IVGHVLDVV...LFRTSLADWIKRY
Cluster-43029.20736   SRTYSY...WEKR.........GVV.CPSKPSL.FVGNFGDVI...LLKKTLGDWIKDL
Cluster-43029.19160   KHKYSY...WKRK.........GVA.YLTPIFP..IGNEPLSL...PKGLSFGLISKKY
```

```
                            70        80        90       100       110
Cluster-43029.107589  FGLGN.NTYEHYNSV.VRAVDETLTKLGATRIGVAGEGDDGAGTMEED..FLAWK....D
Cluster-43029.63569   STTTP.EGKALYQKW.........IIEDNRNIVHILE.............DMPSC....H
Cluster-43029.22743   RDLTQ.KYGSLISTR.LGTQ.LIVVLSDYKMIRDAFRKEE..FTGRP...HNEFT....N
Cluster-43029.10012   WSLRK.QYGKIARVSGLIGHPDLLFIFDGEEIKNVFRQEEAMPHRPSMPSLHYYKQRLQK
Cluster-43029.8125    YSKYT.SP..YFGIY.ILGK.PYLVIRCPEIIKNITIRDFKNFDDRTF..ACDKKA...D
Cluster-43029.14063   YDKMK.GH.RYVGLY.FFSR.KAFLPLDPVLIKDILNKDFQHFSDRGI..YYNEKR...D
Cluster-43029.25266   FKKYN.LYGSLVREEIVPGV.NVVWVFDPNDIETMFRQEGKYPQRRSHLALQKFRLDRPN
Cluster-43029.79453   MNLHL.EYGTFYKLW.LGPR.LHLVIGKPEYLEELLTSNVHLAKSS....GYDLF....K
Cluster-43029.43227   SETYN.SN..VIGLK.LGAE.KLVIVLSYKLIKEVAYAHEYNGRPDNF..FLRIR....S
Cluster-43029.96554   YGKLN.SD..VLRVW.LGPR.LIVGIANAEDAEVILGNSV..HLEKSP..DYSLF....E
Cluster-43029.33848   YLEFK.KRGEKHGGIYYFRT.PVYIPVDPEIIKKIVISDAHYFPNHGF..YINGGY...D
Cluster-43029.78834   EDISK.KLGPLFRLN.LGGI.NLVITTDADNTKTLFRNEGIRPMRPPFAALYYYRK...K
Cluster-43029.88066   YNEFK.LKGLKHGGIYIMTS.PTYLPVDLDIIKNIFQRDFASFMNHGF..YVNEEV...D
Cluster-43029.43879   RNRAK.EFWPIYKLW.SFGL.YTVVLLHPEDIELVLNNT..KHLSKSY..IYSFL....H
Cluster-43029.62226   YNAVP.GV.RFSGMY.QFTT.PTLVIKDPELIKQIGVKDFEYFTDHKN..FIPEEA...D
Cluster-43029.33140   TTLSK.KYGPIYGLN.LGNV.YTVVLTDPKIIKSVFSKDAA..TGRAP..LYLTH....G
Cluster-43029.59200   YG.......SFFRIW.IALL.PTVFIYEPKHLKIIMSTTKNN..QKSL..FYKAL....H
Cluster-43029.76786   .........................................MEIDKEV...........E
Cluster-43029.62259   ..........................LDLEIIKYILVKHFNHFVDRGF..YYNERD...Q
Cluster-43029.74476   YKLFP.NT.RYMGAY.QFTK.PVLLIKDPDLIKDVTVKSFDHFMDHTN..QISQNA...D
Cluster-43029.68359   REWRR.KHGPVYQFT.ALGV.VVVVVSGAEEYEEIVSGLKN..LRKGV..NYELV....E
Cluster-43029.48459   YD.EF.PNTRYSGTY.QFGV.PVLTLRDPELIKQITVRDFEHFVSRRT..YIPEDI...E
Cluster-43029.81172   YD.QF.PESRYSGIY.QFTS.PTLIIRDVDLIKKIAIKDFDCFPDHRI..YTPEAT...D
Cluster-43029.59974   FGKFP.GS.RYFGSY.MFNQ.PSLMIKDVDLLKQMTIRDYEHFNEHRA..FAKEDV...D
Cluster-43029.91416   KLAEM.YNTKVLGLK.LGKE.KVVVVLSYPVVKEVLRREEFEGRPDNF..FIKLR.....
Cluster-43029.69013   EHLRDEFGST.FKIY.TGPLSAVVVSADEQFVEYVLSSQ..NIISKAM..QYEYF....H
Cluster-43029.37757   DKRHR.QLGPIFRDS.VGPV.SAVFLADPDEMRAVFSKEGKYPQHIKPESWLLYNE...R
Cluster-43029.14393   MRYLR.EYGGIVKVH.DGPLSVTLVVTDPAFVKHILSSR..TFIEKSL..QYDFM....R
Cluster-43029.40441   WDKTD.AP..YFGMF.ILDE.PVLVLKSPKLIKDVLVKDFSVFCDRRV..ATPTH.....
Cluster-43029.92595   RDLCK.QFWPIYAMW.TLET.PIVIILHPDDIELVVSNIKHN..EKNF..IYNFV....Q
Cluster-43029.69234   YQRYG.AELRYYGTY.TFNQ.PSFILKDVDLIKQMVIKDFEHFNEHRP..FANEEA...D
Cluster-43029.12188   VTICR.KYPSPFRIW.FGPK.FTVVLTDVEQVQQIVSSSNFITKDPDI...YKFL....E
Cluster-43029.74864   YNSVE.GE.RYYGMY.QFTI.PTLVIKDTELIKQICVKDFEYFVDHRS..FVSSEA...D
Cluster-43029.54028   RRHLE.ANNGLCTFE.ILGS.KVIIASDPTFLEFLLGKT..DILDKST..EYKYL....N
Cluster-43029.76813   YNSVE.GE.RYYGMY.QFTV.PTLVIKDIDLIKQICVKDFEYFIDRRP..FVTSKD...D
Cluster-43029.24503   VELLE.KYGPMIKCYPTLVY.MILCTTDIPLLEHILSSS..RLIDKSE..YYGFI....R
Cluster-43029.14286   EDMYK.KYGPVVKEEALFNI.PVINIFEKSDIEKVFKSSGKYPIRPPTEAMAQYRRSRPD
Cluster-43029.20657   YTKAK.AQGKQYCGY.YDSFIPTLLPIDLELIKNILQNDFSHFTDRWK..HVDVER...D
Cluster-43029.44423   SK.NY.GP..LYRVSLTSLY.PAVNIAGSEELERIICTTKN..IEKGI..VYNML....N
Cluster-43029.64913   YN.KF.PSVRYYGAY.LFNY.PILIVKDPELIKEITVKDFEHFVDHSS..WLPENV...D
Cluster-43029.106926  ...............................MKRDLKKRETNLTSEKF..DTELY.....
Cluster-43029.27430   YEHFK.NEGHKFGGIYDKLN.PVFIPVDEKLVKSILQTDFQNFVNHEG..YVNEKN...D
Cluster-43029.86380   YDELE.HKGCKFGGV.YLGLSPYLVVIDPTYAKDILTKDFQYFTDRVM..YNNSK....D
Cluster-43029.44325   YN.LS.QEARYTGLY.QLTT.PLLLIKDPKLLKQLTIKDFEYFMNHKQ..FVPEEV...D
Cluster-43029.31883   REWAK.KYGPMYALSSTFLF.ASINITGAEEFEKIASTTKN..TKKST..EYIFF....K
Cluster-43029.15394   YDEMD.AR..FFGIY.VFGD.PYLIIKDATLAKTVLIKDFDNFEDRNI..AAPEH....S
Cluster-43029.77583   IKIQK.KYPTLCKVW.LGPR.LIYFVSKPEHIEKILTSNEALNKDN....LYEFL....S
Cluster-43029.58135   YDELE.KRQMKYAGV.TLGTRQILVIRDPDLIKDILTKDFEYFTNRQV..YYQEKT...D
Cluster-43029.26450   YDLSD.EP..YIGIY.VFDV.PVLLLRCPKLIEQVLIKDFSHFRDRSL..ACPKH....N
Cluster-43029.82633   TSYSI.QYGGIIRIY.IPPVRPTVVVACPKIIHLLTTSKYSLNKAK....DYQFF....D
Cluster-43029.62747   YEKEY.DK.PYFGVF.IFDE.PHLILKCPKLVKQIMIRDFINFTDRTV..AYPDH....D
Cluster-43029.65165   DKLHA.KYGKTFKMY.IGGT.MQVLTSDVHLIATLTSKPNPNIIKSNS...YELL....E
Cluster-43029.92705   ..........YYGTY.QFNT.PILLIKDTDLIKRIMIKDFEHFSDHRT..FIPDTV...D
Cluster-43029.11398   ........................MLRCPKLIENVLVKDFGYFRDRSL..ACPKH....N
Cluster-43029.23918   VDEFV.TSPSISTLW.IGPI.LYFRISQPEYIEKVAMNKT...LEKDL..VYQLF....E
Cluster-43029.24230   YDQFK.KMGVKFGGVYIGLD.PYLLIMDPAHAKNIFTKDFQYFTDRGQ..FYSER....D
Cluster-43029.66374   ............................................................
Cluster-43029.27546   YP........LYKMW.CLNL.SAVFLLSPEDIEKVLMSPKN..MQKSF..MYDYL....K
Cluster-43029.36509   YDQID.AP..LVGIF.VLDN.PVLMIKSPKLIHDVMIKDSGNFIDRTT..APSDH....D
Cluster-43029.94585   YDVTN.KK..YMGLY.IFDE.PFFVVRDPEIINNILIKDFDHFVDRNV..AAPKH....D
Cluster-43029.93682   YDQFK.IMGEQFGGI.YLGLDPYLFIMNPQQANRILKDDFQYFENRGI..FYSEK....D
Cluster-43029.106935  YDDTR.ER..YFGLY.IFEE.PIFVPRDAKLIKNILIKDSNHFMDRFI..GHAEH....D
Cluster-43029.20736   YDSTN.ED..FFGVY.IFNS.PLFVPKNPDLIKDILVKKFEYFCDRNV..AAPGH....C
Cluster-43029.19160   YDQFK.PTGCRLGGV.YFGFEPNLVIMDPKYMEDVLKTDFSHFIDRGT..YYNKS....C
```

```
                        120         130       140           150       160
                         .           .         .             .         .
Cluster-43029.107589    PMWAA.....LAESMGLEEREAIYEPTF.AIV...DR...DTLTAESEEVYLGEPNKAYL
Cluster-43029.63569     PALDH...........LCELLPRLQPRYYSIS...SS...PKLYPNTVHI..TAVLVEY.
Cluster-43029.22743     ILGGY..GVINIAGKLWKDQRRFLHDGLRHFG...MSYLSSRKTQMENRI..MFEVEDFL
Cluster-43029.10012     EFFNGNEGVIGVHGPKWEAFRKQVQQFLLPPI...TA...KKYIEPLDII..STEFISRM
Cluster-43029.8125      ELAAN..SLFIMRNPDWKYIRTKLTPIF.TSG...KL...KLMLNIVKES..AKDMQHHL
Cluster-43029.14063     PLSAH...LFSISGPKWKSLRSKLTPAF.SPG...KL...KFMFCTVLRC..GHTMSEKL
Cluster-43029.25266     VYNSG..GLLPTNGPEWFRIRSVFQRGLSSIQ...SV...RRFINSSNNI.....IQEWL
Cluster-43029.79453     PWLGD..GLLVSSGKKWKERRKMITPTF.HFK...IL...EEFMKVFNKQ..FNITLDIL
Cluster-43029.43227     MGTRK..GVVGTDGEIWKIHRHFVVTHLKTLG...FGV..KSMDNMVHEE..VVDILSIL
Cluster-43029.96554     PWLGD..GLLISKGEKWRSHRKMIAPTF.HTS...IL...KSFMPVFNKN..AASLVEAF
Cluster-43029.33848     ILSGH...LFNMEDKEWKILRTTLPSVF.TTA...KM...KKMYMIMQRM..VDPYKEWL
Cluster-43029.78834     AFNST..GVVPSNGEEWYKLRSGVKRLL.DNS...IV...NSYSKQHENI..AQNFVTYI
Cluster-43029.88066     PLTGH...LFALEGEKWKRLRAKLTPTF.TSG...KM...KMMFSTMVSC..TDGLRDIF
Cluster-43029.43879     EWLGT..GLLTSTGKKWHKRRKILTPAF.HFN...IL...QEFLEMLNNE..TDILVDNL
Cluster-43029.62226     QLWGK..NLFSLRGQKWRDMRPVLSPSF.TSS...KM...RAMFMLMSEC..AENFNNHF
Cluster-43029.33140     IMNGF..GLICAEGDIWREHRRFSYNCLRQFGGAKISTQRKKMECLIMEH..VLDFVQYM
Cluster-43029.59200     NFLGQ..GLITNNGNKWKHNRKLIQPFF.HIN...IL...EKFVDLFTEC..SNRFITKL
Cluster-43029.76786     PVFGR..NPFVMKGAEWKNKRAQLTACF.TSG...KI...KGMYAFLEKS..ANRMVQYI
Cluster-43029.62259     PMSAH...LFAIGGTKWKNLRNKFTPTF.TSG...KM...KYMFQTVADC..GISLDKHL
Cluster-43029.74476     PLWTK..NLLSLRGQKWRDMRAILSPSF.TSS...KM...RTMFTLMLEC..SQNFTEHF
Cluster-43029.68359     PWLGQ..GLLTNTGHTWQQRRKILTPAF.HFS...IL...QQFVEIFNKE..TSKLVTVL
Cluster-43029.48459     PLFGK..SLFSLKGQRWRNMRSTISPTF.TSS...KM...KAMFILIKDC..ANKFTNHF
Cluster-43029.81172     PLWAK..NLFAAQGYKWKKMRPVLSPAF.TSS...KM...KMMYNLITRC..AKNFVKYN
Cluster-43029.59974     PMWAK..NLFALTGNRWRQMRSTLSPSF.TSS...KM...KGMFLLMTEN..AQRFIDFF
Cluster-43029.91416     .CMGTRRGVTTTDGPLWRSQRNFVTTHLRNLG...YGR..KPMEMLIIDE..VSDLVSIL
Cluster-43029.69013     NWLGT..GLLTSSGSKWRKRRKMLTPAF.HFS...IL...KNFVGIFASA..EDILIAKL
Cluster-43029.37757     HNYSR..GLFFMDGEEWYRFRKIMNNII........M...KGDLSWIEQS..CEVSTDLL
Cluster-43029.14393     EWLGD..GLLVSTGSKWKQRRRLLTPGF.HFS...IL...EHFVDVFESV..GNIFIEKL
Cluster-43029.40441     PKMIH..SVFFQKYNPWKKIRSKLSPLF.TSV...MM...KNMHVHLEQI..NSRMTDFI
Cluster-43029.92595     AWLGT..GLLTSRGAKWHNRRKILTPAF.HFS...IL...KEFVDVLIRE..TDVLVEDL
Cluster-43029.69234     PMWAK..NLFSLRGNQWRNMRSTLSPSF.TSS...KM...KVMFVLMREN..AQKFVDFY
Cluster-43029.12188     PFNGK..GLITSSGIKWKMDKRLMSSKF.SKK...AI...SQYFLPMLDE..TDKLVDIL
Cluster-43029.74864     PLMSK..NLFALKGQQWRDTRPVVSPVF.TSS...KM...KSMFTLMLEC..SQSFNNYF
Cluster-43029.54028     SWLGS..GLLTAEGPKWKKTRKVLTPSF.HFS...VL...ESFIDVFEAN..GDILIELL
Cluster-43029.76813     TLMSK..NLFALKGQQWRDTRPVLSPVF.TGS...KM...KAMFTLMLEC..SQNFNNYF
Cluster-43029.24503     SWLGD..GLLTSSGIKWKKSRRMLNPGF.HSS...KM...ENFIDVFENV..GDVFIEKL
Cluster-43029.14286     RYAST..GIVNEQGETWYHLRTYLTAGLTSPK...TI...FSFLPQVETI..TDDFCNLI
Cluster-43029.20657     PLNGN...LFSLQSEKWRPIRVQLTPTF.TSG...KM...KMMYQTIIES..IGALQEVL
Cluster-43029.44423     LWLGT..GLLTSTGSKWHTRRKILTPAF.HFN...IL...QQFLTTFHSE..TKTLVDVL
Cluster-43029.64913     PLWSN..NLFSLKGEKWKESRAFFRTAF.TSS...KM...KFMFGLMRDC..AKNYVEFY
Cluster-43029.106926    ...............KW.ALESITTVAL............NRRLGVLDDN..CDENSDAF
Cluster-43029.27430     PLTGH...LFNLRDEKWRNVRAKLTPAF.TTG...KI...KLIFLTMLEA..STGLVELW
Cluster-43029.86380     PFTVH...ILSQTGSDWKNARTKFTSMF.TSS...KM...KNYHESMEKC..TEDLREKL
Cluster-43029.44325     SVWSK..NLISLKDHKWKETRPVLSPFF.SSS...KM...KMMYSLMAEC..SENFVQHY
Cluster-43029.31883     RWLGQ..GLLTSSGSKWQTRRKILTPAF.HFN...IL...QQFLKIFNKE..TNRLVANL
Cluster-43029.15394     RISQS..LLFMQKNPVWRENRTKLTAVF.SSG...KL...KRMLPIFLRS..HENLSKYL
Cluster-43029.77583     LGIGY..GLMTAKAPKWKKHRKAIRPAF.NQK...VL...DDFQQIFANQ..SEIFADIL
Cluster-43029.58135     PISAH...LFSLRGERWRNLRQKLTPTF.TSG...KI...KSMFPALVEL..SKFLVQAF
Cluster-43029.26450     PFIAN..FLLFQHYPEWRKTRSKLNAIF.SIG...KL...KGMYYTSKLV..LVALDDYL
Cluster-43029.82633     EWLGK..GLIFAAVSHWQKNRRLITPYFGRLT...LM...TNFVKIFEKH..GDQLVEHL
Cluster-43029.62747     KVLSN..FLFFMPNPQWKGTRSQLSPTF.TSG...KL...KGMFPIITNV..AEDLQKYL
Cluster-43029.65165     PWLMD..GLILSEGELWKNRRKLCTKSF.HVS...LM...PDFFKTFNAA..ASTLTRKL
Cluster-43029.92705     PLWGK..NLFSLKGQKWRDMRLVLTPSF.TTR...KM...KNMFILMKEC..AENFVRHL
Cluster-43029.11398     SCISS..FLLFQHYPEWRKTRLKLNTIF.SIR...KL...KGMFHTSKHV..LITLDDYL
Cluster-43029.23918     DVVGQ..GLFTAPVKIWKPHRRTIAPCL.NIN...IL...NSYVETFAEC..SNRFVRLL
Cluster-43029.24230     YMAKN...LFTEEGTEWKSIRSKFTHMF.TAA...KL...KNAFGTMKHC..TLDLKKRL
Cluster-43029.66374     ..........................................MMPLIFEC..TNEMTKYL
Cluster-43029.27546     DWLGN..GLLIADHERWQITRKLLNPAF.NFN...IL...QEYVHTFNEE..SQRLVRNL
Cluster-43029.36509     KVSGS..ILFLEKGSKWKKSRENLLPLF.SPL...KL...KRMFPVFEKI..SSKVIGKN
Cluster-43029.94585     NVQSN..MMFFQKNPSWRVVRTRVTPAF.TTI...KL...KAMMHLILEA..SNGMLEFL
Cluster-43029.93682     PLWNN...MFSYNGDEWRRIRPKFTNIF.TPS...RL...KYFFTTMTQC..AENLKEHI
Cluster-43029.106935    DVQSS..ILFFQRSPDWKAMRNKMTPFL.SSG...KN...KAMLPLMLEC..NVEMIEFL
Cluster-43029.20736     EITSH..LLFIQKSPDWKNDRSKVTPAF.TSV...RL...KSMVPLVIAC..AEEMSKYL
Cluster-43029.19160     PLTVN...IFTQPGNEWRATRAKFSNMF.TNS...KM...KILFETFKAC..NEELEEYL
```

```
                           170       180       190       200       210
Cluster-43029.107589  QGTPKGPYNAQNPYTAEVAETRELFNSKDRSCIHMEID...ISGSNLSYQTGDHIA....
Cluster-43029.63569   ..............DTPTGRH...NKGVATTWLKMKI......PDAEKELPTVPIF....
Cluster-43029.22743   FALKA.YDGQTVD.LNPVFAV.SISNVICDVLMSVRFSHN.DRRFIRFMNLIDEGF....
Cluster-43029.10012   EDMLD.TNRELPDHFLSEIYK.WALESVGRVSLNRRLGCL..EPNLSKDSETQKII....
Cluster-43029.8125    SQFDN....EMVE.IKEVSSK.YMTDVVASCFFGCKAKAF.TDKDSEFFNFTKTMF....
Cluster-43029.14063   QELSL..ENGDVE.IKEILAR.YTTDVIGSCAFGIECHCM.SDPDAEFRLMGRRAF....
Cluster-43029.25266   I.............MIETTRN.......................................
Cluster-43029.79453   RKQVNRNPEDSVD.ITENINL.ASLDTICETAFGTSLNA..QYNGNPEYVKALSIF....
Cluster-43029.43227   NESNE....KEIK.IIPIISV.SVLNVLWKSLTGSRLDRNDPNLGELLNLLDKRAI....
Cluster-43029.96554   RREKG....KLFD.CHDNMST.TTVDTLLETAMGVTK....CEEDNTGFDYAMAVM....
Cluster-43029.33848   DKFSE..NREAVE.IKSIVSR.FTFDIISACAFGIDTNTL.SSKNEDFLNHARQFF....
Cluster-43029.78834   RDNLD..NNNVLQDAVEHLMS.FAIEAISIVCPGYRLNCLSDNSHANEIKSASKNF....
Cluster-43029.88066   KSYAK..LQDPMD.IKEVVSR.FTTDVIGSIAFGIDCNSL.KDPESEFRVFGRRIF....
Cluster-43029.43879   KMLCD...APYVD.VLKPITE.FTLYSIGETSLGVPLR...EDPNCLQYKQAIFDI....
Cluster-43029.62226   FQKDK..DVIEVE.MKDIFTR.FTNDVIATTAFGIKIDSL.KESNNEFYLMGKEAT....
Cluster-43029.33140   ELDKN...DQEID.ILPSLRH.NLGSVINLIVFGKSWTR..KDEVWQWLQYLQEEA....
Cluster-43029.59200   NG......ERTVK.ITSFINE.CVLDILHNGVLGVP.....FDKDSPYRQGELQAI....
Cluster-43029.76786   SDETN..PMKPLE.AREVCMR.FTLDNVAACAFGLEGRSF.DDPYSDFRELADKFL....
Cluster-43029.62259   NDEMD..LTKPQD.IKKILGD.FSTDIIGSCAFGIECNSF.TNPENPFRIYGTRVF....
Cluster-43029.74476   LRKDE..KIIEIE.MKDIFSR.FTNDVIATTAFGLKVDSL.QEPENEFYFMGKEVT....
Cluster-43029.68359   KEESK...KPSVN.IVPLLSQ.TTLNTIAETSFGTTLDM..KKQDDKNYVSAIHVL....
Cluster-43029.48459   VEDSN...IVSVQ.FKDTSGR.FCSDVIASIAFGISIDSL.EDKNNDFYTMVSRVT....
Cluster-43029.81172   NSSEG..SLIEVD.TKDLYSR.FATDVIATCAFGIEVNSL.VDRDNGFYHRGCELT....
Cluster-43029.59974   LKQNK..DIVEIE.MKDVSTR.YCTDVIASAAFGLEVDSL.TNANNDFYLKGKELT....
Cluster-43029.91416   KEHEN....EAVG.VGSILAV.SVLNVLWALVSGSR.....LDRNDLRLNRMLELL....
Cluster-43029.69013   EEFSG...EGSFN.IFPFISH.YALDVICEAAMGVPVNAQ.LDGESKYVQNVKSIS....
Cluster-43029.37757   VNNISRQDGREFKNLEEELYK.WSIDVIVSVLVGASSYKECCEDVEGLVKKLASTI....
Cluster-43029.14393   EEEVG...KQSAD.IAELVSL.CTLDVICEASMGIKLDAL..NNKTSAFVRNTKTM....
Cluster-43029.40441   NNNTG.....ELD.VRSIGGH.FATEMIARCFYGVNIFCF.DEEKSLFRKHVHEIA....
Cluster-43029.92595   KKMCD...APYVN.VMKPVTE.FTLNSIGETSLGVSLR...EDPKSLQYKKAVCDF....
Cluster-43029.69234   LNQDK..DLMEIE.IKDAASR.YCTDVIASTAFGLEVDSL.SNPNNEFFIRGKELT....
Cluster-43029.12188   KEKID...KPVFN.IGPYIHR.CAMDYINAIILGIETNAQ.FGELEQFIKVLHRIY....
Cluster-43029.74864   LKQTK..DSKEVD.LKDIFGR.FTNDVIATTAFGLKIDSL.ENPDNEFYVSGKEAV....
Cluster-43029.54028   EKEAA...QDDVD.IFPYCTM.TTLDIICETAMGVSINAQ.RNSQSDYVTSVKEKC....
Cluster-43029.76813   LRQTK..DSTEVE.LKDIFGR.FATDVIATTAFGITIDSL.ENRDNEFYVRGKETV....
Cluster-43029.24503   ENNIG...KDSFD.IFPFTAL.CTLDVICEAALGTKLNA..QDGDSSDYVESVKVM....
Cluster-43029.14286   KQKRQ..KNNIVADLEDVFGR.MGLETSCALVLGHRLGLLLPGEESDFAKVLAEAI....
Cluster-43029.20657   DEKAS..STNPID.IKDILGC.FTSDVIGSVAFGLETNTL.KNPDSDFRKYGKRIL....
Cluster-43029.44423   EKETE...KPWTN.VVPIISL.FTLCAISETSMGATLNM..KSETDRNYITATFKI....
Cluster-43029.64913   KEKDK..DFLEID.LKPSLRH.FTCDVIATTVFGVRANTL.RRPNATFYHMGVEAT....
Cluster-43029.106926  KLISA.........VLKMFHHIYVLDVLQPYWKFVKTKTL............RDFI....
Cluster-43029.27430   KGHAA..SKHPVD.IKNTLAH.FSMDIIASVVFGIESDIQ.INPDSEFKLFTSKLMVPQD
Cluster-43029.86380   KELAD..KNSDVD.IFETIAC.FTTDVISSVIFGIEAHSF.KPSQAVFRKMGRDTF....
Cluster-43029.44325   LKLSKGTDIVDVT.VKEASVR.FSNDIIASAAFGIHIDSL.KEPNNTFFLMGRAIM....
Cluster-43029.31883   EKETD...KYYTN.IVPLVSQ.FTLFSITETSMGTTLDIN.NEEDKNYISAIHQIG....
Cluster-43029.15394   QENRK.....VME.AKELAAK.FATDVISSCAFAIDSKCF.DEKPSDFRKNGRRIF....
Cluster-43029.77583   GKYVG...TKEIN.LFRLVSN.CTLDIICETAMGLSMNL..QTTDCDFGLNMDRLM....
Cluster-43029.58135   DEAAQ..KKGDID.IFEFSAK.FTTDVICSCAFGLESNTF.GDPNSEFRSWSKKIM....
Cluster-43029.26450   KNNLG.....VID.AKDLACR.FSTEIISNCWFGLSSNCF.DTDQSSILAVSRRSF....
Cluster-43029.82633   SNELD...NKDFD.ITSLTKS.YTMDILCETSMGVSTIE..DKYKQKYFKIVHRII....
Cluster-43029.62747   SNNQG.....VLD.AKETVAK.YSTDVIAKCFFGINAHCF.DDENAMFRVLGRAMF....
Cluster-43029.65165   GEAAD..KNAVID.VIQYTSK.MSFDIVAETSFGVKLNS..QDGNHVEYINAIAEY....
Cluster-43029.92705   EDKSE..EIIELD.LKDTFTR.YSCDVIASTAFGIETNSI.QVPKNEFYQTGMELT....
Cluster-43029.11398   RTNPG.....VID.AKELAGK.FAIEIISKCWFGLSSNCF.EDDKSRMFDVYIGTF....
Cluster-43029.23918   KELHG...KQDVD.WKCLTTR.YTFEAFSKTGLGVDIDVISAEESDEDLGNSSKWA....
Cluster-43029.24230   DESYA..KDADVK.IMETLGC.YFTDVVGSAVYGIEAHSF.KRPDAVFRQMGRDGF....
Cluster-43029.66374   LENQR.....VLE.ARELTVK.YSTDVIAKCAFGINAGCF.RNNKAEFRERAKPIF....
Cluster-43029.27546   NEECH...KPYVV.TAELTKN.YSLNCIGSTFIGLQLN...KNINFENYKSILEAY....
Cluster-43029.36509   RNENK......IN.LKEIITI.ISTDVITRCLFGVETDCL..ENDSEFLKYIERLF....
Cluster-43029.94585   HKSQG.....IVD.IKEMAAK.NSTNIIAKCIFGINSHCF.QRDKAPFRENGRPFF....
Cluster-43029.93682   EKHWR..SQEDVY.IQDTFNS.YFTDVIGSVVFGIEANSF.KEPNAVFKRMGNDIF....
Cluster-43029.106935  RKNQG.....ILE.AKDITAK.YTTNVIARCAFGINPKCF...ENECFRDRAKPFF....
Cluster-43029.20736   RKNQQ.....VLY.VDQMTSS.YSIDVISECCFGINPHSF.DRDPTYFTKQGRRIF....
Cluster-43029.19160   AKCVL..NDEYTD.ILNVMSC.YNTDIIGSVVYGVECKSFKDAEDSAFRQIGNSVF....
```

```
                                220        230        240        250        260        270
Cluster-43029.107589  .......VWPSNAGYQVDLFLKALGLTDKRNNVISIKALEPTAKVPFPTPTTYDAIARYH
Cluster-43029.63569   ................IRKSQFRLPTKPQ....TPIIMIGPGTGFAPFRGFVQERN.LYK
Cluster-43029.22743   ...RLFGSLEA...AIFIPVLRYLPRFASTR..QKISRN.....REEMATFLQETI.DEH
Cluster-43029.10012   .NSINTFFWNV......AEVELKLPVWRFY...KNKAFRNYIGALEDFRTLCLKYI.TLS
Cluster-43029.8125    .....SSKLSF...LNLFSYFFAPILVSL....LKLKFL....DYGLLRRIFLDTL.NYR
Cluster-43029.14063   ...TQTIGDL....LKMIIIRSFPPLAGF....LGIGVF.SGGVTKFFNRVVRETI.EYR
Cluster-43029.25266   .................................TNIDFL.....................
Cluster-43029.79453   ...LEVFTLRF.....FSGWLRNPLIFRL....SSLYKK.YTESLKILHGFTNKII.RER
Cluster-43029.43227   .....AFNMAGGTLNTFPWLRHIAP........NKSGYNVIVELNRKLKELLMKTI.EEH
Cluster-43029.96554   ...RMCNILHQ...RHLKFWLRPDVFFKL....TKLSTL.QVGLLDVIHGLTSSVI.KRK
Cluster-43029.33848   ...DYQWKLSK...NAMV.ITIPRKILQA....IKFRIF.PKSTEKFIVETFTSIY.NYR
Cluster-43029.78834   ....MDGLYKT.........LIGPPLWKLY...ETSGYKQLKSSHEFIYRSLDTFL.KTL
Cluster-43029.88066   ...KPTTSQRI...KSLLARMVPRSVLTA....LNYKSI.DTEVEQFFLGVIRSTV.NYR
Cluster-43029.43879   ...GENFQYRA.....VRPWLYIPFIYKL....SDLYRR.DTDIITVLHNFSKNVI.QKR
Cluster-43029.62226   ...NFGGFWKT...LKLLGFFIIPKIYQL....LNVGLF.DKHVSKFFIELIDETI.KTR
Cluster-43029.33140   .....PKYMGVAGPINFLPIFRFVPKYRKI...MSFIKTNQRKTYNVYRELIKEQE.KVL
Cluster-43029.59200   ......KRYIK.......PWLLFESIFNH....TSSAHY.EKRQKDNLYSYTKKIL.INR
Cluster-43029.76786   ...NPDSLRQK...IIFLLFALCPGILAK....LKLKLT.PDEVELRLFELIRGSL.KYR
Cluster-43029.62259   ....AMTPFEN...LKLVFGLNFPKIARF....LRITIM.SEDVNAFFTKMVEDTV.NYR
Cluster-43029.74476   ...NFGGFFRT...FKFILLILFPKVAQA....LKISFI.SKRIRTFFTSLIDNTI.KIR
Cluster-43029.68359   ...GQILVHR....ITRPWWHHN.LLFNLL...SPSAFK.YRKTLNTLLGFTNRII.KQR
Cluster-43029.48459   ...DLTTFWKM...MRFLGFLMVPKLYRL....LGLTIF.DANESQFFVNIVNDSI.RER
Cluster-43029.81172   ...NFSSFWKN...FALFGHLIIPKLYKL....LNLKII.TDDESRYYHNIIEKTI.KMR
Cluster-43029.59974   ...DLRSIYAT...FRFLVNMFASKLAKI....FDIRII.CADVGDYFNEILENTI.KLR
Cluster-43029.91416   .....DKRSRL...FDMSGGVLSQFPWLRFVFPEKTGYNLINHLNTELRQLLNESI.EEH
Cluster-43029.69013   ....QVITQRN.......FSALHPRLYP.....FTINYYKEKMALKVLHAHTDAVI.DRK
Cluster-43029.37757   ..............HKVFKTTCKLQLLP.....ANLAMKYNLSTWKNFENSVKNSL.EAS
Cluster-43029.14393   ...CSIVLHRA.......LSVIHPSIYPF....TYTSFK.EARAIKVLHAYTDKVI.DER
Cluster-43029.40441   .....DFNVRN...AVIQNLFFLKPKWITL...FKLNFI.QEDIMDFFHGVLQYSI.TSR
Cluster-43029.92595   ...GEYITYRA.....TRPWLYLPFIYNR....SPLYKK.VTKATNVLHEFSANVV.NER
Cluster-43029.69234   ...NSQGVKIM...MKFIISIFFPKLMKV....FNINLL.TTEVRDFFTNLIEDTI.KMR
Cluster-43029.12188   ......TIIHI...RILKVWLQQEVLFR.....LSSYWKDHEYGRKFFRNFIEKAIDNTK
Cluster-43029.74864   ...KF.GFIRN...LKFFGYLVVPKLFEI....FKITFM.GKKINKFFTTMIDDTV.KIR
Cluster-43029.54028   ....RLLIERSFSLKRFSILYYLSECYRR...........DVEYVKILHTHTSEVI.TSR
Cluster-43029.76813   ...QF.GFIRN...LKFFGFLLAPKLCEI....LKITFM.GKEINKFFTKMIDDTV.KVR
Cluster-43029.24503   ...CSIFTSRL.......SFPLPPLLYP.....LTMNYYREKKALRILHGRTENII.NRK
Cluster-43029.14286   ...HQNFVAIR.......DTYYGLPIWKYF...ETSAYKKMAESEDTIYKLALELI.RTA
Cluster-43029.20657   ...SMTPRRVM...TYIALGILPSWLTKL....FGLKIF.QKDMENFYINLVRDVV.DYR
Cluster-43029.44423   ...GKLFFYRL.....MRPYLYSDWLYYN....MNSQGRVENKLVNTLHTFTDGII.RKR
Cluster-43029.64913   ...NSSGIRSL...IYLLHFVLPDFLVKL....LNIKIY.SNRVRKFFVTLVENTM.KAR
Cluster-43029.106926  .................................KNSNFL.....TNTISKLIDEGL.AEQ
Cluster-43029.27430   INSKLTFRESMQETFKAFTDLIPEWVWIR....LGVRYI.PKFFEDFLVRVVSDVV.EYR
Cluster-43029.86380   ...AEYSYKRQ...VALFLTLCYPNMARI....MNMGNI.QAHIQDFFLRFVKEAL.EYR
Cluster-43029.44325   ...DFPLLNRG...FKFFAFFMLPKLLKF....LKIEYF.DRISREFFVDVIKRTM.KMR
Cluster-43029.31883   .....PIIFRR....MIKPWLFFDIIHEL....FPSGRL.EIKLVKILHSFTNNII.SKR
Cluster-43029.15394   .....DSNLRN...AFVQYVYFFKPKWAAT...LKLDFL.EKDVENYFCEAFLESM.KSR
Cluster-43029.77583   ....EITTYRI.....FHIWHHLRFTWNL....YPMSKE.YARCLKKFKVFTESIV.RMK
Cluster-43029.58135   ....ELGSLSH...KLSIMLIANAPQLAYN...LGVAAI.RRDLSNYFHNLVANTI.KYR
Cluster-43029.26450   .....AFTFRN...AVVQMLYILKPAWVEF...FNLNFM.EKSDQDYLLNVFKDVV.ESR
Cluster-43029.82633   .....QITLRRMGSAIKRFNFLFILTRD.....AQIYRRDLKVIEDLFEKIVGERIIRQQ
Cluster-43029.62747   .....DFRLRT...AFSQSAYFSMQNFVKW...FRINFF.EAWIIDYFTDSFRKAF.DAR
Cluster-43029.65165   .....TAIVME...KIFSVWKRVRLLFKIF...NSKRYSVQEESLIILNKTAMNII.QER
Cluster-43029.92705   ...TFSGLRSL...IFFVAFFLPDYITKL....LGIRVF.TEKMTKFFTDVIDQTI.KLR
Cluster-43029.11398   .....AFTLRN...AVIQLLYVFKPAWVEF...FKLDFI.KRSDQDYLINVFNTVV.ESR
Cluster-43029.23918   ...YDIGTTKL.....KSLQYYIEPIWTY....SSLKKE.FDKHKDIINKIILSCV.KDK
Cluster-43029.24230   ...YKFSLSDR...IMLFLSVCYPKLLKK....LPLRSIFQHQISEFFEDFVALAV.DHR
Cluster-43029.66374   .....MNTWKN...AIVQAMYFLKPSWLDT...FKLEFF.DKGSIDYFKEIILETM.YNR
Cluster-43029.27546   ...MKSLVYRA...ANPLMMVPYISKW......TRSYRE.GEKIAQKLHQISKTVL.EEN
Cluster-43029.36509   .....SFSWRN...SFAVFSYFSKPELVKI...FKIEFI.ERSAVDYLGAVALEAL.KNR
Cluster-43029.94585   .....SNSLRN...CLIQTLHFLKPSWVEK...LRIGFF.KNSTVRYFESVLLDTM.YNR
Cluster-43029.93682   ...HNLTLVER...FVMFVIMCYPDFLRK....FPIKSIFPERIRVFFVNFVKDTI.EHR
Cluster-43029.106935  .....SSSWRN..AFSQTVYLLIPSLVNK....LKLDFY.EKATTDYFQTIFIDTM.HSR
Cluster-43029.20736   .....DFTFRN...VFSQFIYWFKPSWVNI...LKLEFI.EKEVIDYFSEAFIETL.KTR
Cluster-43029.19160   ...RKHTLSDR...LQMYLALYHQTLARF....LGVTNV.QKGVWEFFLNVLKDSI.EYR
```

```
                                                                280       290
                                                                  .         .
Cluster-43029.107589 MEI.....................................CAPVSRQMVSTLAPFSPTEK
Cluster-43029.63569  EEG.........................................................
Cluster-43029.22743  RKT.....................................F..............DPSHI
Cluster-43029.10012  IEE..............................................IRMNSYHDIQD
Cluster-43029.8125   EKH.....................................N................IKR
Cluster-43029.14063  ERN.....................................D................VVR
Cluster-43029.25266  ............................................................
Cluster-43029.79453  RAE.....................................YKSRGDNNGEHVSEEGIKRR
Cluster-43029.43227  YET.....................................W..............TEGRD
Cluster-43029.96554  KEHYFDRAKRGETTLYGHAVNTTKFDEKDGQIVESRDIMPNYLRDDLDENDENDVGEKKR
Cluster-43029.33848  KQK.....................................G................VNN
Cluster-43029.78834  KNA.....................................H...........DSNPDVLK
Cluster-43029.88066  EEN.....................................N................IFR
Cluster-43029.43879  KET...........................................ISTHKTMSYSNRKR
Cluster-43029.62226  EEK.....................................A................IVR
Cluster-43029.33140  ENL..............................................DTDVNDDDESN
Cluster-43029.59200  KKE.................................................LKDKKITK
Cluster-43029.76786  KDN.....................................K................IVR
Cluster-43029.62259  ESN.....................................D................IRR
Cluster-43029.74476  EEK.....................................R................IVR
Cluster-43029.68359  EEN.....................................F...NTFQVNDDNTLVKSKK
Cluster-43029.48459  EEK.....................................G................IVR
Cluster-43029.81172  KDK.....................................G................VIR
Cluster-43029.59974  EEK.....................................G................IVR
Cluster-43029.91416  YST.....................................................WTKGRE
Cluster-43029.69013  LSE........................................LNYLKKEEISEDGIKNK
Cluster-43029.37757  NAL.........................................................
Cluster-43029.14393  IKR.....................................RETSPKYEVDENTNVKVDKK
Cluster-43029.40441  PNN.....................................G..............NKIEN
Cluster-43029.92595  KKT.............................................FDQGISYSERKK
Cluster-43029.69234  EDK.....................................K................VVR
Cluster-43029.12188  KRN......................................................DKK
Cluster-43029.74864  TEK.....................................G................IIR
Cluster-43029.54028  KEL.....................................KKHTKDELENGKNDVGMKKK
Cluster-43029.76813  TEK.....................................G................IVR
Cluster-43029.24503  MLE.....................................R.SAERAKMGNTNKEDVKKN
Cluster-43029.14286  DEA.....................................T.................QE
Cluster-43029.20657  EKN.....................................N................IFR
Cluster-43029.44423  AEN.....................................F.EMFETNEADEYNYYARKR
Cluster-43029.64913  EEN.....................................G................IVR
Cluster-43029.106926 VDA.....................................S..............VPDHE
Cluster-43029.27430  EKN.....................................N................VYR
Cluster-43029.86380  RKN.....................................N................IYR
Cluster-43029.44325  EES.....................................E................IVR
Cluster-43029.31883  AKT........................................FEAIEYEKENRYMSSKK
Cluster-43029.15394  KDT......................................................ASF
Cluster-43029.77583  IEK.....................................SNAERARKDSLTESFVDGKK
Cluster-43029.58135  KDN.....................................N................KKR
Cluster-43029.26450  RTE.....................................N............RKNKGIA
Cluster-43029.82633  EEE......................................................NEE
Cluster-43029.62747  EKS.....................................Q................QRH
Cluster-43029.65165  REI.....................................N........SKAPEKVSKSK
Cluster-43029.92705  TDK.....................................G................IKR
Cluster-43029.11398  IAA.....................................N............QRNKGVA
Cluster-43029.23918  LQN...............................................CKQQDAPVMKGKQ
Cluster-43029.24230  EKN.....................................N................IHR
Cluster-43029.66374  SDD......................................................VKY
Cluster-43029.27546  KKI.....................................F........EQNINDPNQRK
Cluster-43029.36509  KTR.....................................T................GKS
Cluster-43029.94585  SDD......................................................DRY
Cluster-43029.93682  RKN.....................................N................IHR
Cluster-43029.106935 SHN......................................................DRY
Cluster-43029.20736  KLS......................................................DRR
Cluster-43029.19160  EKN.....................................N................VRR
```

```
                         300       310                                     320
                         .         .                                       .
Cluster-43029.107589  IKTEMTRLGSDKDYFHEKTGSSF............................YNIAQFLTV
Cluster-43029.63569   ...................................................KPVGD....
Cluster-43029.22743   ..RDLLDTYLYEIQKAHEEGTGQDLF.........................EGKDHDRQM
Cluster-43029.10012   QDISILEQILL......KTGN..............................AKL...AAV
Cluster-43029.8125    ..HDFVDLLLQLKDNCKDEDSD.............................IEFDV.DRM
Cluster-43029.14063   ..PDFLQLLIQLRNRGKIDDDVIYESKRPSFS...................SALTM.EEA
Cluster-43029.25266   ..PELSRLFL...............................................................
Cluster-43029.79453   ..VALLDMLMET.....TIDG..............................KELSD.EDI
Cluster-43029.43227   ..DDLIYSFINEM....KKADGEP...........................TTFTE.DQL
Cluster-43029.96554   ..LAFLDFMIEAS....QTKG..............................NVLSD.KEI
Cluster-43029.33848   ..NDLTDTLMKLT....EKDQFRKDYTGEHPI...................KPLDL.NEF
Cluster-43029.78834   EIHPFMHTLFNN.......................................KTLSE.EDR
Cluster-43029.88066   ..KDFMHLLLQLKNRGKVSDDHSIFKTDDDNDPNG................HYLTF.NEV
Cluster-43029.43879   ..LALLDLMLKA.....RNDG..............................EDIDD.DGI
Cluster-43029.62226   ..PDMIQLLIQARKGIQQKEEKAIDTGFAVVNEADL..GKGVQ........EGMTN.IDI
Cluster-43029.33140   EGKNVIQCFILEKQKKAPEIA..............................KNLYHEVQF
Cluster-43029.59200   ..TCFLDMFIEI.....AENN..............................GQFTD.EDV
Cluster-43029.76786   ..NDFLDVVSNL.....PTEN..............................GLFAE.IDI
Cluster-43029.62259   ..NDFMQLLIDIKNNKQEGEQTGEG..........................NSLTI.NEI
Cluster-43029.74476   ..PDMIHLMMEARKGVQYKEENVVSEGFAAVKENVIISGNNSP........TELTN.MDI
Cluster-43029.68359   ..LAFLDLLLNA.....KLTN..............................GIVDD.QAI
Cluster-43029.48459   ..PDVIHLLLQT.....RQENTHEEETTTDEAGFAIVQEQKIEKSKRL...TDITD.EDI
Cluster-43029.81172   ..HDMIHLLMEAKEETNKTLESDKLP.........................MWFSD.EDI
Cluster-43029.59974   ..SDMIQLLVDAK....KGNVKVDEHNNTTDTGFATVAEQL...KAEGN..AQITN.QDI
Cluster-43029.91416   ..DDFIHSYITQMRSDSDPD...............................TSFTE.EQL
Cluster-43029.69013   ..RAFLDLLLGS.....TIDG..............................QPLSR.SDI
Cluster-43029.37757   ..LDVISSKTSNTGLLKRMQD..............................EHLDR.KDV
Cluster-43029.14393   ..LAFLDILLNA.....VSDG..............................EPLSR.RDI
Cluster-43029.40441   L.ADLANKALQDKETG.KADG..............................VEM...ETV
Cluster-43029.92595   ..LALLDLMLKA.....RSEG..............................ADIDD.SGI
Cluster-43029.69234   ..PDMIQLLLEAK....KGDAPIQEHNPVSDTGYATTEEYL...KSDIT..KPITN.EDI
Cluster-43029.12188   F.NPIINQLIEM.....ENKQ..............................YGFCTGNDL
Cluster-43029.74864   ..PDLINVLLEARKSLKQKAEKTSQNGFEEEHKAHL..DQKGV........KEITN.EDI
Cluster-43029.54028   ..KVFLDILLES.....TING..............................KPLTD.TEI
Cluster-43029.76813   ..PDLINVLLEARKSINQKAEKTSQTGFKDEHKTDS..DQKGV........KVITN.EDI
Cluster-43029.24503   ..TSLLDILLES.....TIDG..............................QPLSK.TDI
Cluster-43029.14286   ..SVVFQSVLKA.....DIDD....................................KDK
Cluster-43029.20657   ..KDFLQLLIQLKNHAELEDDCSNSLQKSENSQ..................KGLTL.NEM
Cluster-43029.44423   ..MAFLDLLLNA.....KMSQ..............................GIIDD.NGI
Cluster-43029.64913   ..PDMIHIIMEA.....KKGKSQITEENTIDTGYAIVEADNIYTGSQIKSKMEITN.TDI
Cluster-43029.106926  ..LSVFQRLVKVD....KHVA..............................FVM......
Cluster-43029.27430   ..KDFMHQLIQLK....NMGELTDD..EALTTDSNKN..............IYLTL.QEV
Cluster-43029.86380   ..PDFLQLLIEM.....KDCD..............................DQLTH.EYL
Cluster-43029.44325   ..PDMIHLLLEA.....KRGVQHKDERSISDSSFTTFHEVKLEKPTET...MEMTE.EDI
Cluster-43029.31883   NRLVFLDLMLDA.....KLKD..............................RFIDD.EGI
Cluster-43029.15394   ..NDLIDVLNDLK....SKKE..............................FSSNAEDTA
Cluster-43029.77583   ...YFILLEMHLVSLRLRQLHLIFGAHHSGLESRKQKPKKITAFLDLLIENSNFTE.KEV
Cluster-43029.58135   ..PDFLQSLVELQENTAGSDNA.............................FTL...EQI
Cluster-43029.26450   ..KDYIDILLEATEYQNKNSN..............................SNIGI.TEA
Cluster-43029.82633   ..PTILDMLLKLKSS....................................SSM...KYI
Cluster-43029.62747   ..NDFIDILKDLKIKGEHEGV..............................FEQ...EQL
Cluster-43029.65165   HNSILIDNLMQH.......................................PGIDD.SYI
Cluster-43029.92705   ..PDMIDLLMEASKGQCIQEDENIIDTGFATAKTYIANKNKIASK......FNLSN.VDI
Cluster-43029.11398   ..KDYIDILFEAKENGDEKSN..............................T.IGL.TEA
Cluster-43029.23918   ..VNFLDLILYK.......................................SNFTE.EEK
Cluster-43029.24230   ..DDFLQILMGL.....QNTD..............................LKITL.KEI
Cluster-43029.66374   ..NDLIDIMKDLRKQKEISE...............................VELDD.AKL
Cluster-43029.27546   ..LNLVDILLRA.....KNEG..............................ADMDD.TNI
Cluster-43029.36509   ..QDLIDILKNVEDEL.KMGS..............................KELST.EGI
Cluster-43029.94585   ..DDLVDIMKDLQKTK.KVSD..............................VELDD.AKL
Cluster-43029.93682   ..NDFMELCMEL.....EGTQ..............................LTI...AEI
Cluster-43029.106935  ..NDLIDIMKDFYRDGDASVG..............................TDI...SKS
Cluster-43029.20736   ..NDLVDILNDLKDA..KEID..............................RDGSDRSTV
Cluster-43029.19160   ..MDFVQILIDSS....KSQH..............................ISL...EEM
```

```
                        330       340       350       360       370       380
Cluster-43029.107589  LGEGATWDKIPFSAFIEGLNKLQPRYYSISSSSLVQPK.KVAITAVVETQALPSQDVPFR
Cluster-43029.63569   ........................TVLYFGCRKRSEDFIYEDE............LMEYE
Cluster-43029.22743   QQI...MGDLFSAGMETIKSSLQWAVLFMLHHPDKMLAVQEELDQVIGRQ...R.LPRLE
Cluster-43029.10012   L.....ALDLFLVGVDTTSIAVASTIYQLSQNPDKQEKLFEELKNRLPNV..DS.NVNIS
Cluster-43029.8125    IAQ...SMTFFVAGFETTSNAIAFALYELCLRIDYQDKLRQEIFDHIKTD...D.DITFD
Cluster-43029.14063   AAQ...AFIFFLAGFETTSTTISFAMYEMAVNKSIQDKARNEVDGIFGNC...Q.DLTFE
Cluster-43029.25266   .......................................................ELTGQ
Cluster-43029.79453   REE...VNTFMFEGHDTTASAICYVLYAIAQNPEVQSKLYQEILETVGND..SEKEITMY
Cluster-43029.43227   LMI...CLDIFIAGSQTTSNTLDFAFLMMLLYPDVYEKIISCLNEAFDKS...E.SINFS
Cluster-43029.96554   VEE...VNTIMFEGHDTTAAASSFVLCLLGIHRDIQDKVHAELKELFSDN..INRPITFN
Cluster-43029.33848   SSQ...MFIFLCAGFETSSSTQTFALYELAKNLECQTKLRSEIHRVLAKY..NN.DLTYD
Cluster-43029.78834   TML...AMEVFLGGIDTTAVTVALTMLYLAQNKTVQEKARSSG.................
Cluster-43029.88066   AAQ...CFVFFLAGFETSATTITFALLELALNPQIQDNLYQEIKAVLKKH..NN.EVSYE
Cluster-43029.43879   REE...LDTFIFEGHDTTSISICLTLMVLANEPYIQNEIYEEITSVLGEN...E.HLSFN
Cluster-43029.62226   AAQ...ALIFFFAGFDSVSALMCFMSYELAVNPDIQNRLREEIQETFREC..SG.HLTYE
Cluster-43029.33140   HHL...LADLFGAGLDTTLTSLRWYFMFLAKNPLIQEEIRRELDDVLQDR.....LPSMD
Cluster-43029.59200   IQE...IITFMLAGQDSVGATMAFSMYYIAKYPEIQEKIHKEIFAFDVNG.....KITTA
Cluster-43029.76786   VAH...AASFFGDGYETSSRVMSFLLFNLASNQDIQEKLRQEIIEVVTKN..NN.KVDYE
Cluster-43029.62259   IAQ...SFVFFVAGFETSSTTSTFLLYELARHQGIQDKVREEIHRVLRKY..NG.QITYD
Cluster-43029.74476   TAQ...ALIFFLAGFETVSTTMCFMGYELAANPDYQQRLRIEILEALDEN..NG.KITYE
Cluster-43029.68359   KDE...VNTFMFEGHDTTGTAICWILRQLTIYKEYQDLIYEEILSVVGKA..GN.HPNMN
Cluster-43029.48459   AAQ...CFLFFFAGFESVSTLMCCMAHELAVNQDVQDKLRDEINDAHAKY..DG.NLTYE
Cluster-43029.81172   VAQ...AIVFYFAGFDSVTLLLSYLSYELSIQPDIQDKLRAEIQQTLSDG..KG.EMTYE
Cluster-43029.59974   IAQ...ALVFFFAGYDSSSTLMSYMAYELLVNQDVQEKLRDEIRDTLENC..NH.KLTYD
Cluster-43029.91416   IIV...CLDLFIGGAQTSSNTLDFAFLMMVLRPDIQNKVRACLNESFTKE...E.SINYS
Cluster-43029.69013   REE...VDTFMFEGHDTTSSAIAFTLYCLANHPEVQQKAFQEQKQLFGNS...NVNATVE
Cluster-43029.37757   NRI...IVDLILSSGDTTAYTMAWVLYLISKHKQVQRDLRD...........ND......
Cluster-43029.14393   REE...VDTFIFEGHDTTSSAICFTMFSLAANPIIQEEAFLEQKQIYGGE.RDG.KTTTA
Cluster-43029.40441   VSN...AIFFLIAGQETTSTIISFTLYELAMNQKLQDKLRESIEVNVEKH...G.GLTYE
Cluster-43029.92595   KEE...LDTFIFEGHDTTAVSLSFTLMALANEPKIQEQLHQELISIIGES.....KTPFQ
Cluster-43029.69234   AAQ...ALVFFFAGYDSSSSLMSFLAYELCINQDIQDRLREEIKQTLEDC..ND.KLTFE
Cluster-43029.12188   KDH...LATLYTASEDTITLVISFALVLLGMYTDVQENAVDEIIDVVGDK..NH.KIQED
Cluster-43029.74864   ASH...GMVFFFAGSDAVSNLMCFMAYELAINTDIQNRLRKEIKDTDDDC..KG.MITYE
Cluster-43029.54028   REE...VDTFMFAGHDTTSTAISFCLYCLSTHQDVQKKVVEEQKIIFGTD..KNRRATNR
Cluster-43029.76813   VAH...AMVFFFAGSEAVSNLMCFMAYELAISTDIQNRLRKEIKDTDEEC..KG.VVTYE
Cluster-43029.24503   REQ...VDTFMFAGHDTTATAISFTLYSLANNPEIQEKVYREQIQIFNTDNMKTIRPNAA
Cluster-43029.14286   IAA...IVDFIAAGIHTLKNSLAFLLYLVAKHPGVQEKILEDA.................
Cluster-43029.20657   AAQ...SFVFYMAGFETSATTMTFALLELSQNEDIQKKLRSEINRVLAKH..NN.TLTYA
Cluster-43029.44423   KEE...VNTFMFEGHDTTSMSICFTLMLLANYRKYQDEIYDEIMTVTGDN..EQ.DPDIN
Cluster-43029.64913   LAQ...SLIFFFGGFDTVSTLMRFMAYELAINTVIQDKLREEIQANLSPN..DD.LVSYE
Cluster-43029.106926  ......TLDMILAGIDTTGKSLGAALYYLASNPEKQNKLREELLKYLPEK..ET.VITDE
Cluster-43029.27430   VAH...SFVFFIAGYETTSTAMSFALLYLALDQKMQDKLRSEIRQVLDKH..ND.EITYE
Cluster-43029.86380   TSQ...AFLVFSAGFETSSTTTSLALFELGMHKQIQKKLRTEINTVISKY..NG.KVTYE
Cluster-43029.44325   SAH...TLIFFFAGAESVSSLICFMSHELAVHPEIQIKLREEIVNGFKEC..NG.KLTYD
Cluster-43029.31883   RDE...VNTFLFEGHDTTAMSICFTLMLLANHEHYQQMIYNELMSVYGDF..KD.DPDIK
Cluster-43029.15394   CAL...AITFFLGGYETASTTISFTLYELAKQPKLQDRLRCEIQSMIQNY...G.DLTYE
Cluster-43029.77583   KEE...IDIFLIAGTDTTASVLCCIFTMLGMFQDVQEKVYDEIIDIVGPD...R.SVLPS
Cluster-43029.58135   VAQ...VFLFFIAGFDTSSSAISYCLYELCKNPQMQLKVKEEIDSVLEKY..NG.ELKYD
Cluster-43029.26450   MSN...SLQFFLAGTETMSGLQSFALYELSIHPIIQDKLRKEVIKVVEKH...G.DISFE
Cluster-43029.82633   QNE...VNNFLLGGNDTAAAALAFTIYALSKNPEIQEKVLSEQIDIFGKPDVNS.IVSYA
Cluster-43029.62747   GGA...SMQFFAAGFETTSSAISYTLYELCMNENIQNKLRNEILDHIKDN...D.GITYD
Cluster-43029.65165   LAE...GNNFLFAGQDTTSASLSFISYELSKQPDIQEKLYEEISSYVNGD..IN.NLTQE
Cluster-43029.92705   VAQ...ASIFFLGGLETVATEMRFLAYELAVNFDVQTRLRNEIDATFQKC..EG.NVTYE
Cluster-43029.11398   MSN...TVQFFLAASDAMSGSLSFALYELSIHPKIQAKLRKEVVEVVERH...G.DLSYE
Cluster-43029.23918   LNE...IRTMVTAAVDTTGVAICNILLMLSLYPEVQEEVFEEVVNVVGYE...R.DIENS
Cluster-43029.24230   TAQ...SSLFFFAGFQSSTFTSSLTIFELALHQDFQDRVREEIRSVLEKY..NG.EVVYE
Cluster-43029.66374   VAV...AIQFFLAGFETTSSTISFTLHELSLNKKIQNKLREEINDVIDKF...G.ELTYE
Cluster-43029.27546   RDE...LNTFLFAGHETTAGVLSFFLFSIGNEPEIQEKIYEEIMSIPGDP...D.DPTLN
Cluster-43029.36509   QDILGKALTFFFAGIDTVSYTCSLILYDLTINPHIQVKLRNEIKTKFHDD...D.GITYE
Cluster-43029.94585   AGL...AINFFVAGFETTSNTIAFTLYELSMNKEIQSRLRREVTDAIEKS...G.GFTYE
Cluster-43029.93682   TSN...LTGFFLAGFETSSTSASLTVYELALHPEFQQKVREEIHEVLEKH..NG.EIVYE
Cluster-43029.106935  AGV...AIQYFTAGFETTSSTISFTLLELCFNKEIQDQARQEIDDVIEKF...G.GITYE
Cluster-43029.20736   CGL...AIMFLMAGIDTSSTVITFCLYELALNPRIQEKLRNEIQKTVLKH...G.DITYD
Cluster-43029.19160   TAQ...TFLFFIAGYETASNASSYLLFELALHQDIQQQIREEIIHVLEKY..DN.NIRYE
```

```
                           390       400       410                                 420
Cluster-43029.107589   GVATHFLAALADKHSKRANSDAYDLT........YQITG..........PRARHT.GTHL
Cluster-43029.63569    KKGALTLHLAFSRDQSQKVYVTHLLE.KQADEI.WRIIGD.............GNGHIYI
Cluster-43029.22743    DLTYLPITESTIYEVLRISSIVPMGTTHAPVRD.TKLNG.......FHLPK..HA.HVVP
Cluster-43029.10012    VQENMPYLKACIKETLRMYPVIIANG.RNLQSD.TILAG.......YRVPK..GT.HVIF
Cluster-43029.8125     NIQKMAYLDMVLSETLRRYPFGPFLN.RNCKED.YIIEQTG.....LVVEK..GT.PILI
Cluster-43029.14063    SLMTMHYLDTVIFETMRKYPPAPVFL.RKCTKP.YNVPNSN.....IVIEE..GM.SVLI
Cluster-43029.25266    STLDIRF.NSFTPEERRKYSRSSTLI.KTALNT....................NS.....
Cluster-43029.79453    AINDFKFLDIVVKEAFRMYPPVPIIE.RKLEED.WILDG.......IRVPK..NT.NISI
Cluster-43029.43227    DRARVPYVEAVLYEIQRYCHVIPTSGPKRAVKE.TVLGG.......YTIPK..DT.TILF
Cluster-43029.96554    DTMEMKYLERVILESLRMYPPVPLIA.RKVNED.VKLASGD.....YTIPA..GT.TVVV
Cluster-43029.33848    ALRDMKYLDNCVDEALRMYPILPAIP.RICKEN.YPIPGTD.....FTLEK..GT.LTIV
Cluster-43029.78834    ....EKYLRACIKETLRLSPTAGANS.RFLIKD.TDIGG.......YLIPK.....NVFI
Cluster-43029.88066    AIMDMSYLDKIINETLRKHPPVPGTP.RVCNQT.YRVPGTD.....VILEP..GT.RVSI
Cluster-43029.43879    DLRELKILERCIKESLRLYPSVPVIG.RVAGEE.IHTKTG......YIIPK..NC.NVII
Cluster-43029.62226    AILKMKYMDMVVSEALRKWPNAVATD.RVCTRP.YTIQPTRTGEKPLHIEK..DT.LLWF
Cluster-43029.33140    DLEELPLTEASILEALRIRPVVPVGIPHGTVKD.LEIEG.......FRVPK..GT.MIIP
Cluster-43029.59200    ELNEMKYLEQVIKETLRMAPAVPILS.RVLTED.VTLDD.......KVFPV..GT.NLLI
Cluster-43029.76786    LVHGCQYLDACLSESLRISSVIHHLG.KLCTED.YTYTPTDPKFKKMTVSLKAGQ.PIML
Cluster-43029.62259    ALNEMKYLGQVIDETLRLYPPVPLIT.RDCVED.YIIPGVD.....VKIRK..GT.RVLI
Cluster-43029.74476    ALLKLKYMDMFLSESLRKWPAATLTE.RQCTKP.YTISPQRPDETAVHIEK..GF.TIWL
Cluster-43029.68359    TLNELKLMERFIKETLRLFPSVPFIS.RKLDQD.VVLHG.......YLVPK..EA.NIDI
Cluster-43029.48459    TLVRMKYLDMVISEVLRKWPSQAGAE.RICTKP.YTLQPANKEESPINLDE..GT.VVIF
Cluster-43029.81172    SLLSMNYMDMVVSETLRKWPINVVVD.RMSVRP.YTIEPNSSGEKPVTLEI..GT.VVWL
Cluster-43029.59974    ALVTMQYMDMVVSETLRKWPSNPGMD.RVCTKP.YIIQPVTPEEKPVYLEV..GD.MINM
Cluster-43029.91416    DRSRVPYVEAVLFEIMRFWYVAPLIGPRRVLDD.TTLEN.......YSIPK..DT.TVLL
Cluster-43029.69013    DLNNMKYLEMVIKETLRLYPSVPIFA.REISED.CLWGD.......ITLPK..GI.QILM
Cluster-43029.37757    ..NNTSLLKNVVRETLRLYPVAPFLT.RILPEN.ANICG.......YEVPA..NT.LIVM
Cluster-43029.14393    QLQEMKYLELVIKEALRLYPSVPVLA.RKVPED.MEWEG.......HLLPK..GL.SVVL
Cluster-43029.40441    GIQDNKYMDMCIKETMRKYPALPFLD.RMPVRD.YKLEGTD.....LVIEK..GT.SVII
Cluster-43029.92595    ELGKLKYMERCIKESLRLYPSVPIIG.RVTGEE.IKTKTG......YTIPK..GC.NINI
Cluster-43029.69234    NLNNMKYMDMVVTEALRKWPVMSPIE.RVCTKP.YVIPPTLPHEKEVYLNV..GD.VIEL
Cluster-43029.12188    HLVQLPYIDMIIKEVLRLFPIAGFIV.RKSEKE.TYLND.......YLIPE..NC.SVII
Cluster-43029.74864    ALVKMKYMDMVVSETLRKFPFAVMID.RICTKP.YTISPISENEKPFHIEK..GT.PIVF
Cluster-43029.54028    DLQEMKYLEMAIKESLRLYPSVPYTG.RKASKD.VQYKNG......KIIPK..HA.EIIS
Cluster-43029.76813    VLVKMKYMDMVVSETLRKYPFTVMFD.RICTKS.YTIRPINENEKPFHIEK..GT.TIMF
Cluster-43029.24503    DIQKMKYLDMVIKEALRLYPPVPLLG.RELPRD.KDMEWGG.....NVFPK..GL.NILI
Cluster-43029.14286    ...SKAYSKACVMETFRVLPTANLLA.RVTEDD.LELSG.......YKVKA..GN.VVVC
Cluster-43029.20657    AIMEIDYLEMVILETLRKHPPVSNLA.RVCTKE.YRVADGD.....FVIEK..GT.VVNI
Cluster-43029.44423    QLNEMKLMERFIKESLRLYPSVPYIA.RTLDED.VVING.......HFIPK..GE.MVHI
Cluster-43029.64913    TLLEMKYMDLVVTETLRKWPSGLAME.RICTKP.YTLKAKHLHERDVTLEK..GT.LICM
Cluster-43029.106926   VLSKMHFLTAVLKESTRLAPVALGNL.RTTTKN.IVVGG.......YQIPK..GIDLFTV
Cluster-43029.27430    AILEMKYLEQVILESLRMHPPVGVLT.RICTKD.YNIPNTD.....VVIEK..GR.MVML
Cluster-43029.86380    ALKEMTFLDQTFFETARKYPLVPTLG.RVCVKN.YTFENSD.....VSIEE..GT.GVLL
Cluster-43029.44325    ALMKMKYLEMVVLETLRKWPNFVGTD.RECNKP.YVLRPEKPNEKPLVVEK..GT.VLAL
Cluster-43029.31883    DLNELKIMERCIKESLRLYPSVAFIA.RTLDDD.MFLKG.......YILPK..KS.VVHI
Cluster-43029.15394    AINDMKYLDKCIQETLRMYPVLSFLD.RRCKTD.YRIENSD.....VIIEK..DT.PVLI
Cluster-43029.77583    DLPKMKYTERFIKETLRIFPVAAFFA.RSITSD.IDAGD.......VVFPA..GS.TAFF
Cluster-43029.58135    ALAEFKYLEQVIDETLRLYPPLVNLD.RTCVKD.YKMRNGK.....GTIGK..GA.VILI
Cluster-43029.26450    SLRDMEYLECCICETLRKYVSLQIID.RKAVED.YNVPGTD.....FIIKK..GT.VVYI
Cluster-43029.82633    DLQKMKYLEMVINESLRMYPPIPFIG.RKMDKD.IVLDNG......VVLPK..NL.NVVI
Cluster-43029.62747    SVLNLSYLDMCVKETLRKYPVIPFLD.RKCLND.YKIPGTD.....LLIEK..GI.PVYI
Cluster-43029.65165    IVSEMKYLDMVVREALRIRTPIPFIQ.KQVIKD.TLIGD.......TMYPK..GT.TISI
Cluster-43029.92705    VIMKMNYLDMVVSESLRKWPSGVNLE.RVCTKS.LRIPALQSHEKDIIIEK..GT.LVWL
Cluster-43029.11398    SLQDMKYLECCIFETLRKYVALQILD.RKAVED.YVVPGTD.....FVIEK..GT.VVYI
Cluster-43029.23918    DLPSLKYTEMVIKECLRLLPVVLFFS.RIVHED.LDLGD.......IVLPG..GS.NVAV
Cluster-43029.24230    AIGEMNLLSQAWHETTRKYPPLPTLN.RVCVKETYTFENSD.....LTIEK..GT.PVII
Cluster-43029.66374    SVKELRYLDCCIKETLRKYPGLPFLE.RKCDKD.YPISDTD.....IVIKK..GM.PVFI
Cluster-43029.27546    QLQELKYTGLCIKECLRLYPPVPFVS.RVAGED.IKTKSG......YIIPK..GC.NLVL
Cluster-43029.36509    SINCLKFLDMCVSESLRKYPLVTHVC.REARSN.YTFAGTD.....ITIEK..GT.SVYI
Cluster-43029.94585    SLKDMHYLNACINETLRKYPTLSFLD.RRCNTN.YRVPGTD.....VVIEK..GT.GVFI
Cluster-43029.93682    AIGEMQLLTQAWQETVRKYPVVTTLN.RSCTKD.YTFSNSN.....VTIKS..GT.KVLI
Cluster-43029.106935   AIKEAKYLDFCISETLRKYPVSPFLA.RTCTRD.YQIPDSD.....LVLDK..GT.AILI
Cluster-43029.20736    GIQEMLYLDMVFLETMRKYPVISFLD.RKCMKD.YKIPGTN.....VIIEK..DT.AIVI
Cluster-43029.19160    TLKEMPLLDRAYWETLRKYPASGTIL.RQCVKD.YTFRDSN.....LTIPK..GM.KVLC
```

```
                          430       440       450       460       470
                              .         .         .         .         .
Cluster-43029.107589  PVHIRHSNFKLPSDPSRPIIMVGPGTGVAPFRAFVQERAKQAQDGAE.......VGKTLL
Cluster-43029.63569   ............................................................
Cluster-43029.22743   LLHAVHMNPSLWEEPEK................FSPNRFINSEGKVH.......KPEYFL
Cluster-43029.10012   PHLVVSNVEEYVSQPEK................FIPERWLKFDHPECPIHQEKIHPFVSM
Cluster-43029.8125    PLDGLHYDPEYFPNPEV................FEPDRFM..DGNKHNYS....QSCVYM
Cluster-43029.14063   PCYGLHRDPEFFPEPDL................FDPERFS..EENKT...KI..WDGTYI
Cluster-43029.25266   CILKTDNGPQLWKN...................FDTPLYK..KLKRS...QEYMEENIYW
Cluster-43029.79453   FIYGMNHDPNVFPQPEK................FDPERFH..PERQS...TR..HAFGFI
Cluster-43029.43227   SFYSIHQDKAFWKDPEV................FRPERFL..DTD.....GKLSVPEQFV
Cluster-43029.96554   GQFLIHRNKKYWTNPNK................FDPDNFL..PERCQ...SR..PYYSFI
Cluster-43029.33848   SNMGIQRDPEYYPNPLQ................FDPERWT..YENKL...NR..PFVANM
Cluster-43029.78834   ............................................................
Cluster-43029.88066   PFQAIHRDPEYYPDPDR................FDPDRFS..DENKA...NR..HPCAFI
Cluster-43029.43879   DIYGMHR.SRIYENPEK................FDPDRFL..LENVT...NR..HPFSYL
Cluster-43029.62226   PIIGLHRDPNIYPDPDR................FDPERFN..DENKG...NI..DPYTYI
Cluster-43029.33140   LQWALHMNTRVWKNPDE................YNPKRFINEEGKVE.......KSEYFM
Cluster-43029.59200   SPFITQRLAHVYPSPQK................FDPDRFE..ESNVE...KM..HPYAFL
Cluster-43029.76786   PVEGLAMDPLYFDEPQK................FDPERFI..SNGNI.......NKFTYF
Cluster-43029.62259   PIRGIHYDEEYYANPEV................FDPERFS..EENKQ...SR..NQYSHI
Cluster-43029.74476   PVFAIHRDPKYYANPEK................FDPERFN..EENRV...KI..NPYTYF
Cluster-43029.68359   WIYDIHRDPKHWPNPTK................FDPDRFL..PENCV...DR..HPFAFV
Cluster-43029.48459   PTFGLHRDPNFFPNPEK................FDPERFS..DENKH...QI..IPYSYI
Cluster-43029.81172   PTYALHHDPQNYAHPEQ................FDPERFS..EANKT...KI..RSGTYL
Cluster-43029.59974   PIYGIHHNPELFPDPET................FDPERFS..NENKS...NI..KPCSFQ
Cluster-43029.91416   HVHSIHFDKEYWGDPDV................FRPERFLDSDNKII.......FHERFL
Cluster-43029.69013   VPYMIQRSAKYYHNPLE................FIPERFL..DIDGK.......NPYRYI
Cluster-43029.37757   SIYTSGRSDEYFKNSSS................FLPQRWN..RDKKEDLP....MQQASM
Cluster-43029.14393   STYAMHRDPENFPEPNK................FIPERFE..NANLT.......NPFIYT
Cluster-43029.40441   PFFALHRDEKYYPEPEK................YIPERFE..DETINS......DGLTFI
Cluster-43029.92595   HIFDMHRHPELWENPDQ................FDPDRFL..PDNCS...KR..HPFAYL
Cluster-43029.69234   PVFGIHHDPELFPDPEV................FDPERFG..DENKL...NV..KQGSFQ
Cluster-43029.12188   SIYNIHRDSRYWERPDQ................FYPEHFL..PEAVL...KR..NSFAYM
Cluster-43029.74864   PVWGIHRDPKYYADPER................FDPERFS..DENKD...DM..NPYAYI
Cluster-43029.54028   VIYVANRNPKYFPDPDK................YDPDRFQ..ENAQT.......LPYTYL
Cluster-43029.76813   PIWGIHRDPKYYADPER................FDPERFS..DENKG...DM..NPYAYL
Cluster-43029.24503   PIYAIQRNPKYFPEPTK................FIPERFE..DSNGT.......HPYVHI
Cluster-43029.14286   HTGIACKNESYFDNATE................FRPERWL..NEERSS..TTYNSTFLVT
Cluster-43029.20657   PVYCIQRDPEYYPDPDR................FDPERFN..EENKA...AR..HFAAYL
Cluster-43029.44423   HIFDIHRNTKDWSDPEK................FDPDRFL..PENCV...DR..HPFAYV
Cluster-43029.64913   PTIGLHRDPEYYPNPDV................FDPERFS..DKNKA...NI..KPYTYF
Cluster-43029.106926  HLHSSTMTDKYIKEPTK................FLPERWL..RSVNDEYSSKNIHQFTQL
Cluster-43029.27430   SVNGIHNDSDIYPHPQV................FNPDRFS..SENKN...KI..PPTAFL
Cluster-43029.86380   PVLGYHRDPKYYPDPLK................WDPSRFD..NKNE........KHQGYF
Cluster-43029.44325   PIMALHYDPQYFPDPQR................FDPERFS..DENKK...NI..DPYTYF
Cluster-43029.31883   WIYDIHRDPKYWPDPEK................FDPDRFL..PENCI...NR..HPFAYI
Cluster-43029.15394   PLYGYHRDGKYFPRPHT................FDPDRFAVKTSGS........ERAYFL
Cluster-43029.77583   GPVHIQRNPKYWPDPLK................FDPDRFL..PENVS...KR..HPCTSI
Cluster-43029.58135   SLVGIHRDAEFYPDPEK................FDPERFS..AENKK...ER..HPYLHI
Cluster-43029.26450   PLHALHMDEKYYPNPEE................FIPERHQ..KNVEND......GVFNFM
Cluster-43029.82633   SIYGCHHDPKHFPEPDK................FQPERFL..SDI.........IPCSFI
Cluster-43029.62747   PLFALHYDSKYFPEPNR................YNPERFL..NTNFNS......DGLVYI
Cluster-43029.65165   LLYWLHRDSEVYPDPEK................FDPERFS..PENQN...NR..SSFAFL
Cluster-43029.92705   PIMGIHYDPNYYTDPNK................FDPERFN..ENNRSLL.....NDYNYV
Cluster-43029.11398   PFYAIHMDEKYYPNPQE................FIPERYQ..ENVVND......GGLNFI
Cluster-43029.23918   IIYLVHRNPTHWKDPSK................FDPYRFS..PEEVA...KR..HPCAYI
Cluster-43029.24230   PVLGFQRDENIYPDPLR................WDPDRFA..DKDV........KYEAYY
Cluster-43029.66374   SVYGLHLDEKYFPDPYR................YDPDRFT..DGKDN.......HKLAFL
Cluster-43029.27546   SIFDVHRSPEIWEEPDK................FKPDRFL..PENCK...GR..RSFAHI
Cluster-43029.36509   PLYGLHMDEKYFPNPTE................YDPYRFE..KSHC....ND..GQLYYL
Cluster-43029.94585   PSYGLHMDANYFPNPQK................FDPERFR..TKVNG.......SKPIYL
Cluster-43029.93682   PILGYHRDEKLYPDPKI................WDPKRFA..DKNV........KHEAYF
Cluster-43029.106935  PAYGLQMDSTHYPDPQK................YDPERFR..NFSKD.......FGLTYV
Cluster-43029.20736   PVYGLHRDEQHFPDPNK................FDPDRFA..VKS.........QEMCYL
Cluster-43029.19160   PLFSYHRDPDYFPDPVR................WNPDRFL..NPEA........KHLGLL
```

```
                         480       490       500       510       520       530
                           .         .         .         .         .         .
Cluster-43029.107589    FFGSRNKKVLGDKFELVTAFSRDTAKKVYVQHRLKEKGEHVHELLQKKALFYVCGDAARM
Cluster-43029.63569     ........CGDAKSMATDV..RAILIKILREKGSMVEEQATAYLKKMETQKRFSAD....
Cluster-43029.22743     PFGVGRRMCLGEILARMEL..FLFFSSLLHCFDISVPEGERLPTLKGIAGITISPN.A..
Cluster-43029.10012     PFGYGRRSCLGRRFAEVEL..HILLAKIFRRYKVEYHYGP....LTYKITPTYVPEQP..
Cluster-43029.8125      PFGMGPRNCIGDRFGLICA..KIGIVYFLRKYKVEKCKETPVPLVLDPRSPFMVPLNG..
Cluster-43029.14063     PFGDGPRNCIGMRFAMIQA..KIALSLTLRNFYFELSEKTKLPLRMESKGIILTPIGG..
Cluster-43029.25266     EY....RNV............HLWLFTQNLKFSLLLC.......................
Cluster-43029.79453     PFSAGPRNCVGQRYAVYQL..KTCIIKFMLEFEMMEDSNFRPEIGM...CSVLKSRNG..
Cluster-43029.43227     PFGFGK...........NL..KM.LSSINQKLN...........................
Cluster-43029.96554     PFSAGPRSCVGRKYAMLKL..KVLLADVLRKFEIVSLDQEKD..FKLQGDIILKREEG..
Cluster-43029.33848     PFGEGPRMCVGKRFGLVQT..KLGLASIIKDYDVTLSDKTENGFKFVYSELILRKTGD..
Cluster-43029.78834     ............................................................
Cluster-43029.88066     PFGEGPRICIGARFGLLQA..KVGLTAIIRDFEVNLNKKTKVPIKYSTKAFVTAVDGD..
Cluster-43029.43879     PFSAGSRNCIGQRFAIMEL..KICLSGILKYFKLEPVTKPED..IIYKSDLVLRVKGE..
Cluster-43029.62226     PFGVGPRNCIGSRFALLET..KVVFYHMLLNFELVPSPKTKVPLKISTKTFNLVAEGG..
Cluster-43029.33140     PFQIGKRMCVGDELARMLL..FLFASTIVKKCSFSLKDENIS..TWGECGITLTPPDH..
Cluster-43029.59200     PFSLGPRNCIGYKFALIEL..KTMLFYILRKYQLTLPPGKDK..LIFSYRATLRATGG..
Cluster-43029.76786     PFGEGQRACLGQRFATTQI..KVGLVHILKNFKLTVNSKTQFPLKIDPYYIMRHVVGG..
Cluster-43029.62259     PFGEGPRICIGLRFGMMQV..KVGVIHILKNFKVTLNKKTTVPLKYEPISFIPKIVGD..
Cluster-43029.74476     PFGLGPRNCIGSRFALLEI..KVLFFSLLQHFEIVPVETSKIPLTISRNNINLSSEGG..
Cluster-43029.68359     PFSAGPRNCIGKVF.........GISYI................................
Cluster-43029.48459     PFGSGPRGCIASRLAILET..KIFFYYLLLKCDIVPIEKTCIPIRLIQRPSVIAIEDG..
Cluster-43029.81172     PFGMGPRTCIGSRFVLMET..KIFLFYLLFNFEIVPVSRTVIPVKVSTSTFNMKPEKN..
Cluster-43029.59974     PFGLGPRNCIGSRFALLET..KILFFYILKQFKIIPVDRTRIPPEWPKTIFTLIPTDG..
Cluster-43029.91416     PFGFGKRRCLGEILARSCI..FTFFSEIIRKYQILPISEAEKPTGIPTFGITLTPEK...
Cluster-43029.69013     PFSAGPRNCIGQRFAMLEV..KSIVARVLRRFELLPG.DQKIKLELIPE.LILTSKNG..
Cluster-43029.37757     PFGIGARSCVGRKIAETQL..QMTLSKIVKNFEVEILNERDIEDIL...SMVSKPSEP..
Cluster-43029.14393     PFSAGPRNCIGQKFAMLEM..KSIISKVLRNFELLPA.TPTHELQLSPETILVSKN.G..
Cluster-43029.40441     PFGEGPRKCLGRRLGMIAV..AMALSNVISLYRVEKCSKTPETIEFEPKNFALLSKVG..
Cluster-43029.92595     PFSAGSRNCIGQKFAILEL..KIALCGIVKNFKLEPVTKPED..IIYKSDLVLRPSNE..
Cluster-43029.69234     PFGLGPRNCIGSRYALLET..KILFFYILNHFKIVTTDKTVIPVQLPKSVFTLIPTEG..
Cluster-43029.12188     PFSAGPRGCIGKHLAMMAI..KIMLVTILQNFTIQSVGKVED.LKLKTDISVRPKDER..
Cluster-43029.74864     PFGVGPRNCIGLRYALLET..KVLFYKFLSNFEIVPGPKTRIPLKLSSKQLNVAPEGG..
Cluster-43029.54028     PFSAGPRNCIGQKFAWNVM..RSIISKVLRNYEVLPCGHE....IVLSAEIVLKSKNG..
Cluster-43029.76813     PFGAGPRNCIGLRYALLET..KILFYQFLSNFEITPGTKTKIPLKLSSKLFNVAPEGG..
Cluster-43029.24503     PFSAGPRNCIGKKFAMLEM..KSIISKVIRNFELRPS.HPCVDLKLVADVVLTSID.G..
Cluster-43029.14286     PFGIGKRICPGKRFIEQVL..PMILENVVRNFEIETV..GPMELQFE...FLLSPKGP..
Cluster-43029.20657     PFGEGPRICIGLRFGKLQA..KIGLCSVLRNYKVTLNEKTQLPITYETG.FISSVKNG..
Cluster-43029.44423     PFSAGPRNCIGQKFAMLEL..KAVLYGILKNFILEPV.DTPDTVVLVPDMVLRTNHEL..
Cluster-43029.64913     PFGVGPRNCIGTRFAIIEA..KVLFFYLLKTFEIVPVPNSKIPIKIRKSNFHLDGN.G..
Cluster-43029.106926    PFGFGPRSCIGRRIAKLEL..EIATANIIRNFELSWN....QPEMTFRGKFLYGFDDP..
Cluster-43029.27430     PFGEGPRFCIGSRFAIVQA..KLGLCQLLRNFEVTLSEKTQIPITYEEKLHFPTVKGG..
Cluster-43029.86380     PFGDGPRNCIGSRLGLMQV..KLGLAVILLDYEVSVNPSTETPLELDELTFVLKTKNP..
Cluster-43029.44325     PFGLGPRYCIGSRFALLEI..KALFAHLLKSFEIVPTKKTAIPIILAKSNVNNYPVDG..
Cluster-43029.31883     PFSAGPRNCIGQKFAMLEM..KAVLCGILKNFILKPV.DTPETIVLKSDVVLRTKDTK..
Cluster-43029.15394     PFGAGPRSCIGGRFGKLTT..KVGIISFLLNSRFTTCEATPDPLVFSTKSTLLQSTVG..
Cluster-43029.77583     PFSYGPRNCIGAMYAMMNV..KTVLGTVLRRYKMYTDYKSVDEIRLKTNIVLRMKDGPK.
Cluster-43029.58135     PFGDGPRNCIGKRFGVMQT..KIALIQILRNFRLTVSPSTK.KLTYKSHGLFLKPKHP..
Cluster-43029.26450     PFGAGPRICIGERFALMIT..KMMLASLMLSYEVEPCDRTCIPVRLETKSFSFQSVGG..
Cluster-43029.82633     PFSNGPRNCIGRKFGLLLL..KSSIAKIIRNFHILPA.DTDYEMKLFP.SLVLATTNG..
Cluster-43029.62747     PFGDGPRSCIGERFGLLST..KLGLIYILTKFEVKPCEQTPKYIEFEPRSLVLQSKLG..
Cluster-43029.65165     PFSAGGRNCIGQKYALLEI..KTALSRIIYEFTIHPTKNFKLELGL...GAILRSKHG..
Cluster-43029.92705     PFGVGPRNCIGSRYAIMEM..KVLFFHLLRKFEIIPVEKSKIPIEILKRSFTLDGD.C..
Cluster-43029.11398     PFGAGPRQCIGRQFAMIKS..KMMLASVMLSYEVEPCARTPIPLKLEPKSFTFQPIGG..
Cluster-43029.23918     PFSYGPRNCIGWQYAMINL..KTIISTIVRQMKVSTAYASLEEVQI.SWGFIMSVANG..
Cluster-43029.24230     PFGDGPRNCIGLRFGLLQV..KLGLATVLRDYKVTISPKTKLPLKFDPLTFLTSPD.V..
Cluster-43029.66374     PFGDGPRGCIGERFGRLSV..KLALISIIRHFELEKCDNSPDPVEFDPKCILLTSIGG..
Cluster-43029.27546     PFSVGSRNCIGQKFAILEL..KAAIYSIIRNFKLE.SKHTPEDIRLFSNVVLRTKDIY..
Cluster-43029.36509     PFGNGPRVCIGERFALILM..KIVIIQFLLNFELEKCPATPEKIEFELKSYFMRTKEQ..
Cluster-43029.94585     PFGNGPRKCIGERFGLLTV..KISIVRIITKFELDKCESTPDSIKFDPRCLIFTSATD..
Cluster-43029.93682     PFGEGPRSCIGSRLGLLQV..KLCLATLLRDYEVKISPKTKVPLKLDPLAFNSSPK.D..
Cluster-43029.106935    PFGDGPRSCIGERFARLII..KVAVMDILRTFELKECVNSPNPIHFDPKCFLLTPKTG..
Cluster-43029.20736     PFGEGPRACIGNRLGSITG..KVALITILLNYEILKCKETPVPVEYEKKSFLVCVKSG..
Cluster-43029.19160     PFGDGPRNCIGMNFGTTQV..KMGAISVLKNYKITADPNIKLPLEFDPDTFLLYSTQK..
```

```
                    540       550       560       570       580
Cluster-43029.107589 AREVNTTLAQIIAEHRKITPAKADEVVKSMRAANQYQEDVWS
Cluster-43029.63569  ..........VWS.............................
Cluster-43029.22743  ..........FKVKLTPRPVAWESEESNLRPAGSH.......
Cluster-43029.10012  ..........LKFKLLRREM......................
Cluster-43029.8125   ..........LKMNIKKI........................
Cluster-43029.14063  ..........MWLDLRPRHQPN....................
Cluster-43029.25266  ..........YLIVI...........................
Cluster-43029.79453  ..........IKIRLKPRSRN.....................
Cluster-43029.43227  ..........................................
Cluster-43029.96554  ..........FRIKVVERA.......................
Cluster-43029.33848  ..........VWLNLRKIK.......................
Cluster-43029.78834  ..........................................
Cluster-43029.88066  ..........VWLTAQERK.......................
Cluster-43029.43879  ..........LKVKFIPRSKM.....................
Cluster-43029.62226  ..........FWFNMKRLTK......................
Cluster-43029.33140  ...........KFIVKQIS.......................
Cluster-43029.59200  ..........VWITLTPR........................
Cluster-43029.76786  ..........LWLDFEKLV.......................
Cluster-43029.62259  ..........IWLDLKKIE.......................
Cluster-43029.74476  ..........FWFGLKRIKS......................
Cluster-43029.68359  ..........................................
Cluster-43029.48459  ..........CWLGLKCISNIKE...................
Cluster-43029.81172  ..........IWLGFKRCNNNS....................
Cluster-43029.59974  ..........FIMGLQRV........................
Cluster-43029.91416  ..........YKARFIQVK.......................
Cluster-43029.69013  ..........IHLSLKDRRQNGFK..................
Cluster-43029.37757  ..........LRLVFGKIQ.......................
Cluster-43029.14393  ..........VRIALKKRVY......................
Cluster-43029.40441  ..........LPLKITAIVKNTNKY.................
Cluster-43029.92595  ..........LRVKFVRRS.......................
Cluster-43029.69234  ..........INMGLKRI........................
Cluster-43029.12188  ..........FPVRLRLR........................
Cluster-43029.74864  ..........FWVVIRRINQ......................
Cluster-43029.54028  ..........IRVKLRKRDWEKI...................
Cluster-43029.76813  ..........SWLLIKRIKQ......................
Cluster-43029.24503  ..........IPLSLIRR........................
Cluster-43029.14286  ..........TSMIFRDRV.......................
Cluster-43029.20657  ..........VWLNLEKI........................
Cluster-43029.44423  ..........LKVKFCKRHDTN....................
Cluster-43029.64913  ..........WIFGFRKLE.......................
Cluster-43029.106926 ..........LIFTVKEL........................
Cluster-43029.27430  ..........LWLNVEATS.......................
Cluster-43029.86380  ..........VLIRLTKI........................
Cluster-43029.44325  ..........INLGFKPLR.......................
Cluster-43029.31883  ..........LRMKFCKRST......................
Cluster-43029.15394  ..........MPLMFNPLD.......................
Cluster-43029.77583  ..........VWIERR..........................
Cluster-43029.58135  ..........VYLLADKI........................
Cluster-43029.26450  ..........LPLRFKLINTVKLK..................
Cluster-43029.82633  ..........VRISLKRRKIVC....................
Cluster-43029.62747  ..........LMMKFVELPSA.....................
Cluster-43029.65165  ..........FPVKLHRRQ.......................
Cluster-43029.92705  ..........FWFGLKRIYPK.....................
Cluster-43029.11398  ..........LPLRFKPITTIKEQ..................
Cluster-43029.23918  ..........YKLSFQPRENIHSR..................
Cluster-43029.24230  ..........IYVNLTKVD.......................
Cluster-43029.66374  ..........LPIRFKQIIK......................
Cluster-43029.27546  ..........LQVKFIPRK.......................
Cluster-43029.36509  ..........IYVGLRPL........................
Cluster-43029.94585  ..........LFIKFEPLTHQINSN.................
Cluster-43029.93682  ..........IFVNLQKIR.......................
Cluster-43029.106935 ..........LYIQFTEITHQI....................
Cluster-43029.20736  ..........LPLQFKPLVS......................
Cluster-43029.19160  ..........LKLKLHKLDE......................
```
