# Supplementary material for: Molecular Docking of Detoxification Enzymes from Oides leucomelaena with Volatiles of Star Anise
Source: Biology (Basel). 2025 Oct 14;14(10):1411. doi: 10.3390/biology14101411 (PMC12562175; doi:10.3390/biology14101411)
Supplement: Supplementary file 1 [file Supplementary Figure S2.pdf › Supplementary Figure S2.pdf]

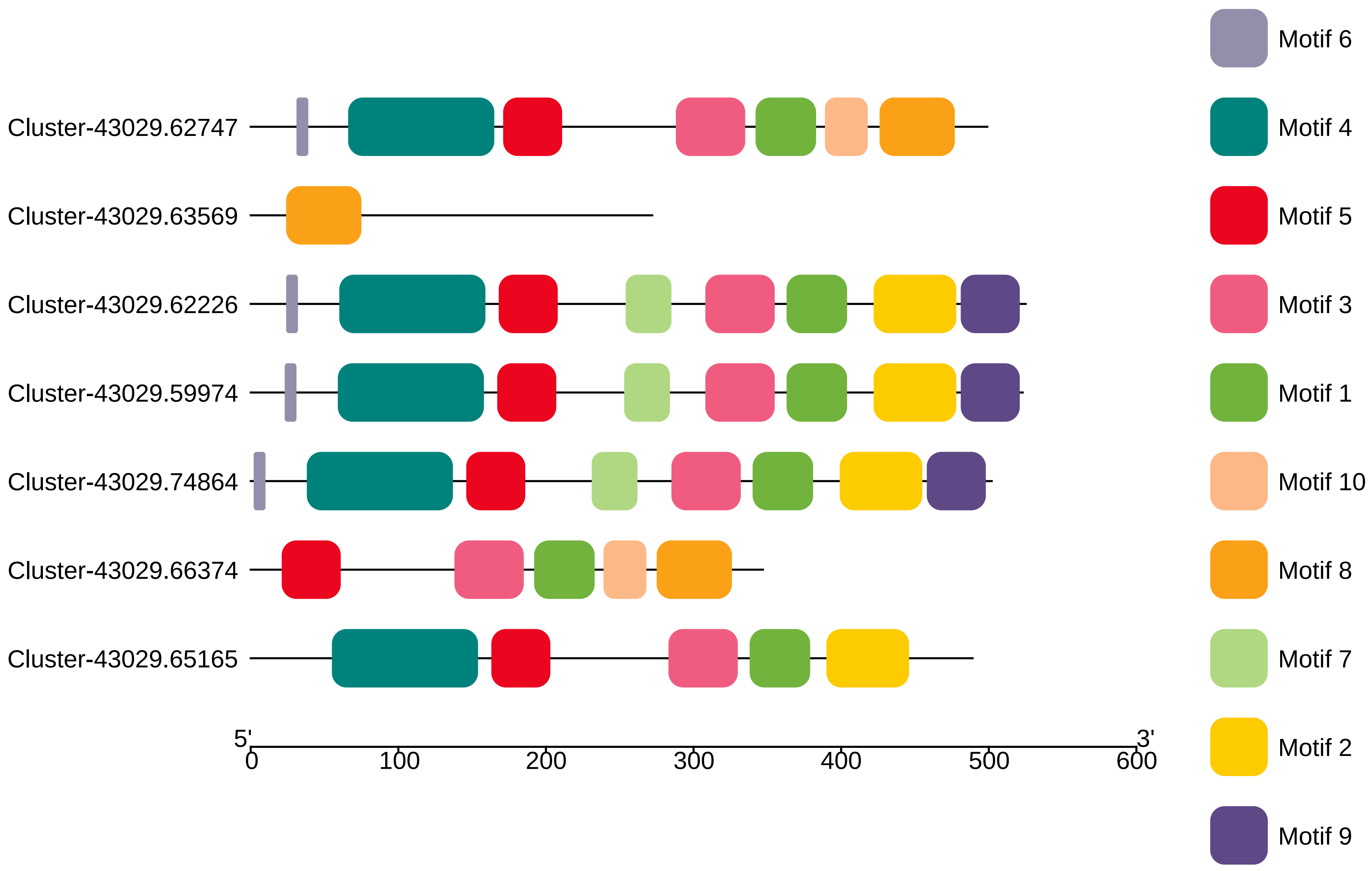
Cluster-43029.62747
Cluster-43029.63569
Cluster-43029.62226
Cluster-43029.59974
Cluster-43029.74864
Cluster-43029.66374
Cluster-43029.65165
5'
3'
0
100
200
300
400
500
600
Motif 6
Motif 4
Motif 5
Motif 3
Motif 1
Motif 10
Motif 8
Motif 7
Motif 2
Motif 9
